# Loss of NR2F6 Protects from *Salmonella* Typhimurium Infection

**DOI:** 10.1002/advs.202404280

**Published:** 2025-07-02

**Authors:** Johannes Woelk, Christa Pfeifhofer‐Obermair, Julia Benz, Natascha Brigo, Milena Bamberger, Alexeja Kleiter, Martin Hermann, Guenter Weiss, Natascha Hermann‐Kleiter

**Affiliations:** ^1^ Institute of Cell Genetics Department for Genetics Medical University of Innsbruck Innsbruck 6020 Austria; ^2^ Department of Internal Medicine II (Infectious Diseases, Immunology, Rheumatology, Pneumology) Medical University of Innsbruck Innsbruck 6020 Austria; ^3^ Department of Anaesthesiology and Critical Care Medicine Medical University of Innsbruck Innsbruck 6020 Austria; ^4^ Present address: Institute of Microbiology – Clinical Microbiology, Immunology and Hygiene University Clinic Erlangen Friedrich‐Alexander‐University Erlangen‐Nuremberg 91054 Erlangen Germany; ^5^ Present address: Laboratory for Langerhans Cell Research Department of Dermatology Venereology & Allergology and 3D Bioprinting Laboratory Department of Pediatrics I Medical University Innsbruck Innsbruck 6020 Austria

**Keywords:** Orphan nuclear receptor NR2F6, phagocytosis, red pulp macrophages, *Salmonella* Typhimurium infection, Sirpα, tissue‐resident macrophages

## Abstract

Nuclear receptors regulate key functions of mononuclear phagocytes and are critical components of the innate immune system, acting as regulators of organ health and disease. In healthy mice, the loss of the nuclear orphan receptor NR2F6 alters tissue‐resident macrophage populations in the liver, lung, and spleen. In response to *Salmonella* Typhimurium infection, *Nr2f6*‐deficient mice exhibit improved clinical outcomes, characterized by reduced weight loss, bacterial loads in the spleen and liver, and decreased plasma pro‐inflammatory cytokines. Despite unchanged basal iron metabolism in the spleen and liver, iron regulatory proteins and the interleukin (IL)‐6‐hepcidin axis are altered in *Nr2f6‐*deficient mice during *Salmonella* infection, reducing hypoferremia. Transcriptomic analysis of splenic red pulp macrophages reveals significant alterations of phagocytosis‐related genes, including upregulation of signal‐regulatory protein alpha (Sirpa). In vitro, phagocytosis of red blood cells, regulated by the inhibitory CD47‐Sirpα axis, and *Salmonella* Typhimurium phagocytosis are significantly impaired in *Nr2f6*‐deficient splenic macrophages. Blocking Sirpα in vitro restores the phagocytic activity of *Nr2f6*‐deficient macrophages to wild‐type levels. In vivo*, Salmonella* Typhimurium loads are partially increased post‐infection in anti‐Sirpα treated *Nr2f6*‐deficient mice. These findings uncover a previously unrecognized role of NR2F6 in host‐pathogen interactions, positioning it as a potential therapeutic target for infectious diseases.

## Graphical abstract

Loss of nuclear receptor NR2F6 reduces tissue‐resident macrophage populations. *Nr2f6*‐deficient mice are protected from weight loss and bacterial load during infection with *Salmonella* Typhimurium. Pro‐inflammatory cytokines and iron levels are altered in infected *Nr2f6*‐deficient mice. Enhanced Sirpα levels reduce phagocytic uptake in *Nr2f6*‐deficient splenic macrophages. Blocking Sirpα partially adjusts phagocytic responses of *Nr2f6*‐deficient macrophages to wild‐type.

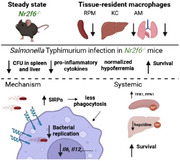

## 1 Introduction

Tissue‐resident macrophages (M_TRs_) originate from distinct lineages and self‐renew within tissues, independent of hematopoiesis. Conversely, short‐lived monocyte‐derived macrophages continuously arise from hematopoietic stem cells in the adult bone marrow and predominantly migrate to inflamed lesions.^[^ ^1^, ^2^, ^3^, ^4^ ^]^ Thus, the constitution of tissue‐resident macrophage populations during fetal development and the role of adult hematopoiesis in maintaining steady‐state levels are organ‐specific.^[^ ^3^, ^5^ ^]^

In homeostasis, splenic red pulp macrophages (RPM) and liver‐derived Kupffer cells (KC) play vital roles in the clearance of red blood cells (RBC) and for re‐utilizing iron to maintain a balanced homeostasis but also for the immediate elimination of microbes.^[^ ^6^, ^7^ ^]^ Receptor‐mediated phagocytosis of RBCs involves Stabilin‐2, Tim‐4, CD300; Fc‐receptor C3; or CD36, conversely CD47–Sirpα interactions serve as an inhibitory mechanism for macrophage phagocytosis.^[^ ^8^, ^9^ ^]^ During blood‐borne infections, the capacity of RPMs and KCs to control iron availability is pivotal for host survival.^[^ ^10^, ^11^ ^]^ Dysregulation of M_TR_ functions contributes to various diseases, such as cardiovascular and metabolic pathologies, obesity, cancer, and infections.^[^ ^5^, ^12^ ^]^

The gram‐negative bacterial pathogen *Salmonella enterica serovar* Typhimurium (*Salmonella* Typhimurium; STM) is a prevalent cause of human foodborne disease and is a crucial model for bacterial pathogenesis.^[^ ^13^ ^]^ *Salmonella* Typhimurium is capable of entering macrophages through both active invasion—mediated by the Salmonella Pathogenicity Island 1 (SPI‐1) type III secretion system—and through phagocytosis by professional phagocytes such as macrophages.^[^ ^14^, ^15^ ^]^ As an intracellular pathogen, *Salmonella* Typhimurium invades various cell types during the early stages of infection, primarily localizing within tissue‐resident macrophages and mononuclear phagocyte populations in the spleen.^[^ ^16^, ^17^, ^18^, ^19^, ^20^, ^21^ ^]^

Generally, phagocytosis is a host defense mechanism by which macrophages engulf, contain, and destroy pathogens. However, *Salmonella* Typhimurium is an intracellular pathogen that exploits this process to gain entry into host cells, particularly macrophages. Once inside, *Salmonella* can replicate within a specialized compartment called the Salmonella‐containing vacuole (SCV), which avoids lysosomal fusion and replicates.^[^ ^20^, ^21^, ^22^ ^]^ This intracellular lifestyle protects the bacteria from extracellular immune effector mechanisms employing complement, antibodies, or neutrophil extracellular traps (NETs). Therefore, despite macrophages serving as the primary defense against bacterial invaders, they paradoxically act as a crucial niche for the colonization of *Salmonella* Typhimurium.^[^ ^20^ ^]^

*Salmonella* Typhimurium requires iron for DNA synthesis, respiration, and oxidative stress defense. It expresses multiple iron acquisition systems, including siderophores, therefore, host‐mediated limitation of bacterial access to iron can limit their growth.^[^ ^22^ ^]^

Further, cellular iron levels also determine the efficacy of anti‐microbial host responses and immune cell differentiation.^[^ ^23^, ^24^ ^]^ Activated macrophages must precisely balance their inflammatory responses to control pathogens while minimizing inflammation‐induced tissue damage efficiently.^[^ ^25^ ^]^ However, the molecular mechanisms underlying this delicate balance remain incompletely understood.

The orphan nuclear receptor subfamily 2, group F, member 6 (NR2F6, EAR2, COUP‐TF III) is a member of the nuclear receptor (NR) family, which controls both pro‐and anti‐inflammatory processes.^[^ ^26^, ^27^ ^]^ Within the NR family, particularly the peroxisome proliferator‐activated receptors (PPARs) PPARα, PPARδ, and PPARγ, in conjunction with the liver X receptor (LXR)α and Nur77 (NR4A1), govern critical aspects of macrophage and monocyte development, functional responses, metabolism, and host defense pathways.^[^ ^5^, ^21^, ^27^, ^28^, ^29^, ^30^, ^31^, ^32^, ^33^ ^]^ During *Salmonella* Typhimurium infection, specific NRs, including PPARδ,  PPARα, estrogen‐related receptor γ (EERγ), and LXR, play essential regulatory roles in infection control.^[^ ^28^, ^31^, ^34^, ^35^ ^]^ Recent findings identify NR2F6 in human macrophages within the regulatory transcription factor cytokine network.^[^ ^36^ ^]^ In mice, deleting the myeloid‐Toll‐like receptor (TLR) 4 induces a reparative macrophage subset (Nr4a1^+^Nr2f6^+^) with significantly upregulated anti‐inflammatory and tissue repair‐related signaling.^[^ ^37^ ^]^

We explored the functional role of NR2F6 during myelopoiesis in the bone marrow of *Nr2f6*‐deficient mice recently, the granulocyte‐monocyte progenitor (GMP) populations are decreased, while monocyte‐dendritic progenitors (MDP) are increased, resulting in altered non‐classical monocytic population in the blood and reduced neutrophil and macrophage populations in the spleen.^[^ ^38^, ^39^ ^]^ Herein, we explore the impact of NR2F6 loss in germline‐deficient mice on tissue‐resident macrophage subset identities and their functional properties during homeostasis and bacterial infection.

## 2 Results

### 2.1 Loss of NR2F6 Alters Splenic Macrophage Populations

**Figure 1:**
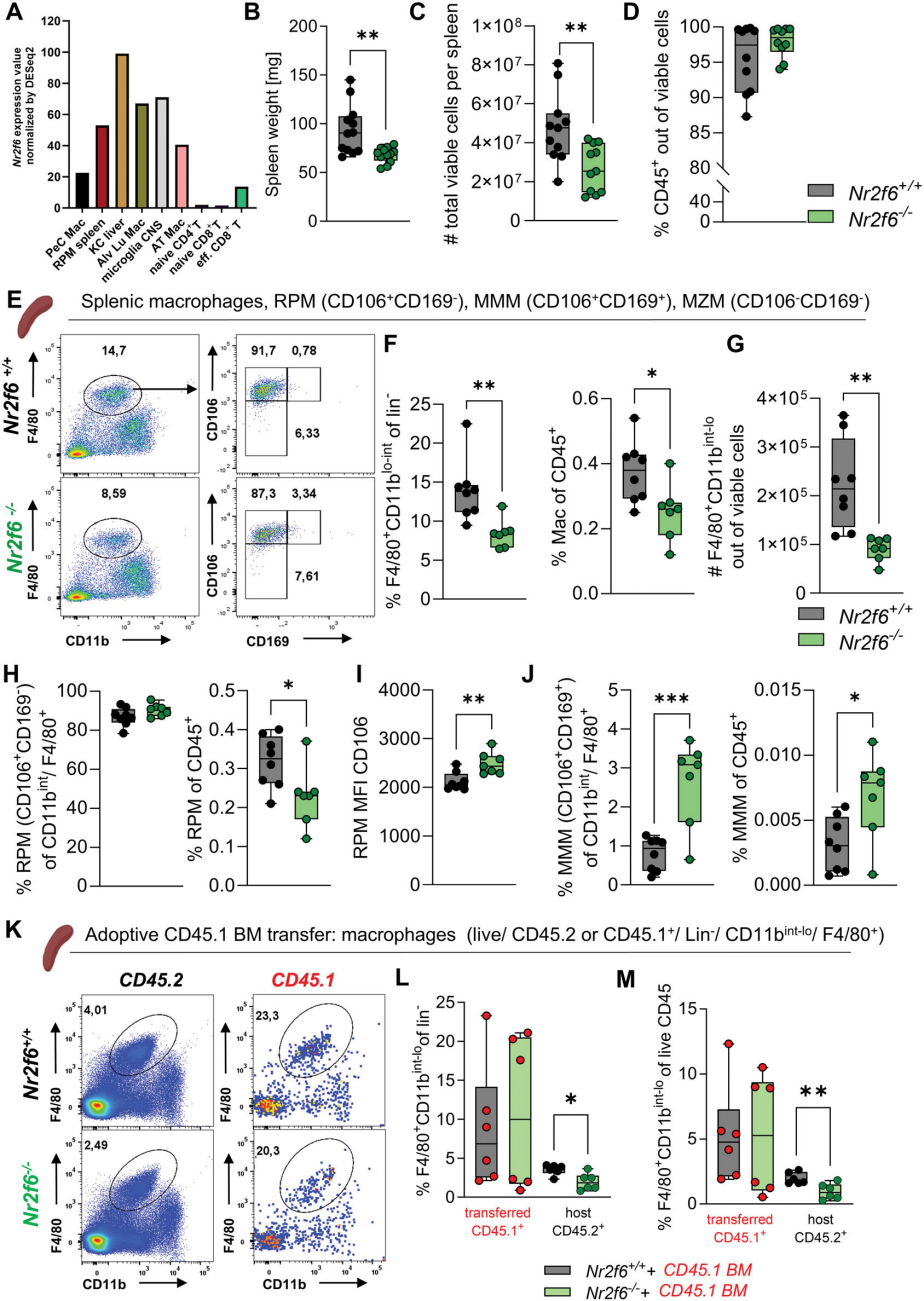
Loss of red pulp macrophages in *Nr2f6*‐deficient spleens. A) Relative *Nr2f6* expression in different tissue‐resident macrophage populations such as peritoneal macrophages (PeC Mac), splenic red pulp macrophages (RPM spleen), liver Kupffer cells (KC liver), lung alveolar macrophages (Alv Lu Mac), microglia in the brain (microglia CNS), and adipose tissue macrophages (AT Mac) compared to naïve CD4 (naïve CD4^+^ T), CD8 (naïve CD8^+^ T) T and effector CD8 T cells on day 7 post lymphocytic choriomeningitis virus infection (eff. CD8^+^) as defined by the Immgen consortium.^[^ ^40^ ^]^ B) Splenic weight of adult wild‐type (*Nr2f6^+/+^*) or *Nr2f6*‐deficient (*Nr2f6^−/−^*) mice. C) Quantification of total viable cell numbers and D) total cell numbers of CD45^+^ cells of adult wild‐type (*Nr2f6^+/+^*) or *Nr2f6*‐deficient (*Nr2f6^−/−^*) spleens. E) Representative dot‐plots of splenic macrophages (CD11b^int^/ F4/80^+^) and subsequent gating on red pulp (RPM: CD106^+^CD169^‐^), marginal metallophilic (MMM: CD106^+^CD169^+^) and marginal zone macrophages (MZM: CD106^‐^CD169^‐^). F) Quantification of frequencies of CD11b^int^/ F4/80^+^ macrophages of lin^‐^, frequencies of CD45, G), and total cell numbers of adult wild‐type (*Nr2f6^+/+^*) or *Nr2f6*‐deficient (*Nr2f6^−/−^*) splenic macrophages (CD11b^int^/ F4/80^+^). H) Quantification of frequencies of RPMs within CD11b**^int^**/ F4/80**^+^** macrophages, frequencies of CD45, and I) MFI of CD106 of wild‐type (*Nr2f6^+/+^*) or *Nr2f6*‐deficient (*Nr2f6^−/−^*) splenic RPMs. J) Quantification of frequencies of MMMs within CD11b^int^/ F4/80^+^ macrophages and frequencies of CD45 wild‐type (*Nr2f6^+/+^*) or *Nr2f6*‐deficient (*Nr2f6^−/−^*) splenic MMMs. K) Adoptive cell transfer of bone marrow cells (CD45.1) into either wild‐type (*Nr2f6^+/+^*) or *Nr2f6*‐deficient neonatal pubs to allow wild‐type (CD45.1) tissue‐resident macrophage seeding into the spleen during perinatal development. Representative dot‐plots of host (CD45.2) wild‐type (*Nr2f6^+/+^*) or *Nr2f6*‐deficient (*Nr2f6^−/−^*) splenic macrophages (CD11b^int^/ F4/80^+^) or transferred bone marrow‐derived macrophages (CD45.1) transferred perinatally on day P4 and analyzed in the spleen of adult mice. L) Quantification of frequencies of CD11b^int^/ F4/80^+^ macrophages within lin^‐^ and M) frequencies of CD45 host (CD45.2) macrophages (CD11b**^int^**/ F4/80**^+^**) or macrophages (CD45.1) derived from bone marrow cells adoptively transferred into wild‐type (*Nr2f6^+/+^*) or *Nr2f6*‐deficient (*Nr2f6^−/−^*) neonatal pubs. Representative data shown are from at least three independent experiments with *n* = 2‐4 per group and experiment, total *n* = 8/8 (*Nr2f6^+/+^*)/(*Nr2f6^‐/‐^*) for adult macrophage populations, *n* = 6/6 for adoptive transfer experiments. Each dot represents the data from one individual mouse. Results are shown as median ± IQR with whiskers from min. to max. The Shapiro‐Wilk test evaluated the normality of data. Asterisks indicate statistically significant differences between genotypes calculated using the Student's *t*‐test or Mann‐Whitney *U* test for non‐parametric data. A *p*‐value < 0.05 was considered statistically significant,**0.01, ***0.001, ****0.0001 (See also Figure S1, Supporting Information).

Data analysis from the Immgen database revealed elevated *Nr2f6* expression in various tissue‐resident macrophage (M_TR_) subsets, emphasizing its probable functional importance. Compared to naïve CD4^+^ or CD8^+^ T cell subsets and even effector CD8^+^ T cells, following lymphocytic choriomeningitis virus (LCMV) infection, *Nr2f6* expression is notably higher in the peritoneal cavity, splenic red pulp, liver, lung, brain, and adipose tissue macrophages (**Figure** **1**A).^[^ ^40^ ^]^

To determine M_TR_ populations in *Nr2f6*‐deficient mice during homeostasis, we examined the frequencies and overall numbers of the red pulp (RPM), marginal metallophilic (MMM), and marginal zone macrophages (MZM) in *Nr2f6*‐deficient mice (8–12 weeks) together with the expression levels (MFI) of CD106 and CD169 (Figure S4A,B Supporting Information). The weight of the spleen and total splenic cell numbers were significantly reduced in *Nr2f6*‐deficient mice (8–12 weeks), whereas the frequencies of viable CD45^+^ cells were comparable to wild‐type (Figure 1B–D). In accordance with our recently published data, the splenic macrophage population (CD11b**^int^**/ F4/80**^+^**) was reduced in *Nr2f6*‐deficient mice (Figure 1E–G).^[^ ^41^ ^]^ Loss of NR2F6 did not affect the percentage of RPMs within CD11b**^int^**/ F4/80**^+^** macrophages, but reduced the percentage of CD45^+^ RPMs (CD11b**^int^**/ F4/80**^+^**/CD106^+^CD169^‐^) in the spleen, the MFI of CD106 was significantly enhanced (Figure 1E,H,I). In contrast, both the percentage of MMMs (CD106^+^CD169^+^) within CD11b**^int^**/ F4/80**^+^** macrophages and the percentage of splenic CD45^+^ MMMs was significantly enhanced (Figure 1E,J), resulting in significantly reduced total RPM but unaltered MMM cell numbers in the spleens of *Nr2f6*‐deficient mice (Figure S1C,D Supporting Information). The percentage of MZMs (CD106^+^CD169^+^) within CD11b**^int^**/ F4/80**^+^** macrophages and the percentage of CD45^+^ MZMs and total MZMs cell numbers were reduced in the spleens of *Nr2f6*‐deficient mice compared to wild‐type (Figure S1E–G Supporting Information). Viability measurements via 7‐AAD/Annexin V staining and spatial distribution analysis revealed no significant differences between the genotypes (Figure S1H,I Supporting Information). To identify intrinsic versus extrinsic factors influencing the macrophage niche in the spleen, we adoptively transferred congenic bone marrow from CD45.1 mice into either wild‐type or *Nr2f6*‐deficient neonatal pubs, according to Scott et al., investigating the seeding properties into the spleen.^[^ ^42^ ^]^ In adult mice within the host macrophage populations, we observed significantly reduced percentages of parent lin^‐^ and live CD45.2 cells within *Nr2f6*‐deficient mice when compared to wild‐type controls. In contrast, the macrophage population derived from the adoptively transferred CD45.1 cells did not differ between spleens of wild‐type or *Nr2f6*‐deficient mice (Figure 1K–M).

Taken together, loss of NR2F6 alters the splenic tissue‐resident macrophage compartment and significantly reduces RPM and MZM cell numbers in the spleen of *Nr2f6*‐deficient mice.

### 2.2 Liver Kupffer Cell and Alveolar Macrophage Populations are Altered in *Nr2f6*‐deficient Mice

**Figure 2:**
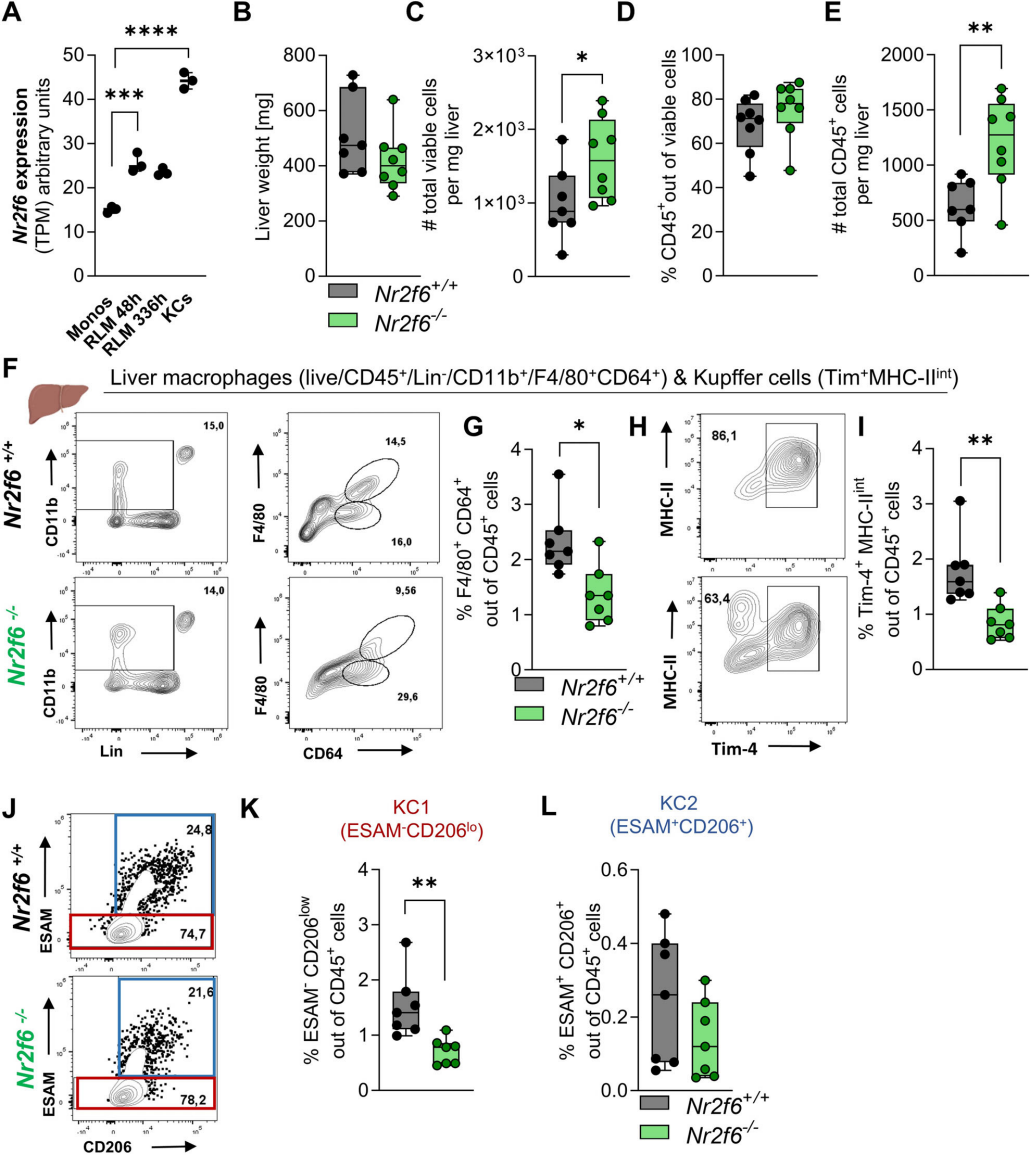
*Nr2f6*‐deficiency leads to a reduction of Kupffer cell frequencies. A) *Nr2f6* gene expression measured by RNA‐seq (TPM) for circulating monocytes, recruited liver monocytes (Monos), repopulating liver macrophages (RLMs), and resident Kupffer cells (KCs).^[^ ^43^ ^]^ B) Quantification of liver weight and C) total viable cell numbers per mg of liver in adult wild‐type (*Nr2f6^+/+^*) or *Nr2f6*‐deficient (*Nr2f6^−/−^*) mice. D) Quantification of the percentages and E) total CD45^+^ cells per mg of liver in wild‐type (*Nr2f6^+/+^*) or *Nr2f6*‐deficient mice. F) Representative dot‐plots and G) quantification of frequencies of liver‐resident macrophages in wild‐type (*Nr2f6^+/+^*) or *Nr2f6*‐deficient (*Nr2f6^−/−^*) mice. H) Representative dot‐plots and I) quantification of frequencies of liver‐resident Kupffer cells in wild‐type (*Nr2f6^+/+^*) or *Nr2f6*‐deficient (*Nr2f6^−/−^*) mice. J) Representative dot‐plots and quantification of frequencies of liver‐resident K) KC1s and L) KC2s in wild‐type (*Nr2f6^+/+^*) or *Nr2f6*‐deficient (*Nr2f6^−/−^*) mice. Representative data shown are from at least three independent experiments with *n* = 1‐2 per group and experiment, total *n* = 7/7‐8 (*Nr2f6^+/+^*)/(*Nr2f6^‐/‐^*). Each dot represents the data from one individual mouse. Results are shown as median ± IQR with whiskers from min. to max. The Shapiro‐Wilk test evaluated the normality of data. Asterisks indicate statistically significant differences between genotypes calculated using the Student's *t*‐test or Mann‐Whitney *U* test for non‐parametric data. A *p*‐value < 0.05 was considered statistically significant, *0.05,**0.01, ***0.001, ****0.0001 (See also Figure S2, Supporting Information).

To determine if the loss of the splenic macrophage population in *Nr2f6*‐deficient spleens is organ‐specific or if NR2F6 might exert a broader influence on M_TR_ populations, we extended our investigation to evaluate macrophages in the liver and lungs. Recent RNAseq data indicate enhanced *Nr2f6* expression transcript per million (TPM) in Kupffer cells compared to recruited liver monocytes or repopulating liver macrophages (**Figure** **2**A).^[^ ^43^ ^]^ Kupffer cells modulate iron homeostasis in mice and are essential for recycling damaged or senescent red blood cells.^[^ ^7^, ^44^ ^]^ We isolated *Nr2f6*‐deficient hepatic leukocytes following perfusion and determined the proportions and overall counts of M_TR_s and KCs following the methodology outlined by Bleriot ^[^ ^45^ ^]^ (Figures S1A and S2A Supporting Information). We observed no changes in liver weight, but viable liver cell counts increased in *Nr2f6*‐deficient mice (Figure 2B,C). While the frequency of *Nr2f6*‐deficient liver lymphocytes (CD45^+^) remained unchanged, the cell count per milligram of the liver increased due to the elevated number of viable cells when compared to wild‐type controls (Figure 2D,E).

In contrast, the relative percentages of total liver macrophages, particularly Kupffer cells (Tim^+^ MHC‐II^int^) and KC1 (ESAM^‐^ CD206^lo^), were significantly reduced in *Nr2f6*‐deficient mice (Figure 2F–K; Figure S2A Supporting Information). Notably, the KC2 (ESAM^+^ CD206^+^) population remained unchanged (Figure 2L). However, due to increased viable cell numbers in *Nr2f6*‐deficient livers, total cell counts per milligram of liver tissue remained unaltered compared to wild‐type (Figure S2B–F Supporting Information). The gross spatial distribution of F4/80^+^ cells within the liver was consistent between the genotypes (Figure S2G Supporting Information).

Concurrently, lung analysis on *Nr2f6*‐deficient and control mice was performed to investigate alveolar lung macrophage populations, employing the protocol outlined by Liu.^[^ ^46^ ^]^ Lung weights, live cell counts, and CD45^+^ cell counts per milligram lung were unaltered between genotypes (Figure S2H–J Supporting Information). However, percentages and absolute counts of alveolar macrophages were significantly reduced in *Nr2f6*‐deficient mice (Figure S2K–M Supporting Information). Thus, the loss of NR2F6 strongly reduced splenic macrophages and impacted M_TR_ populations within the liver and lungs, albeit to a lesser extent.

### 2.3 Intrinsic Loss of NR2F6 in Bone Marrow‐Derived Macrophages Alters M1 Responses and Phagocytosis In Vitro

**Figure 3:**
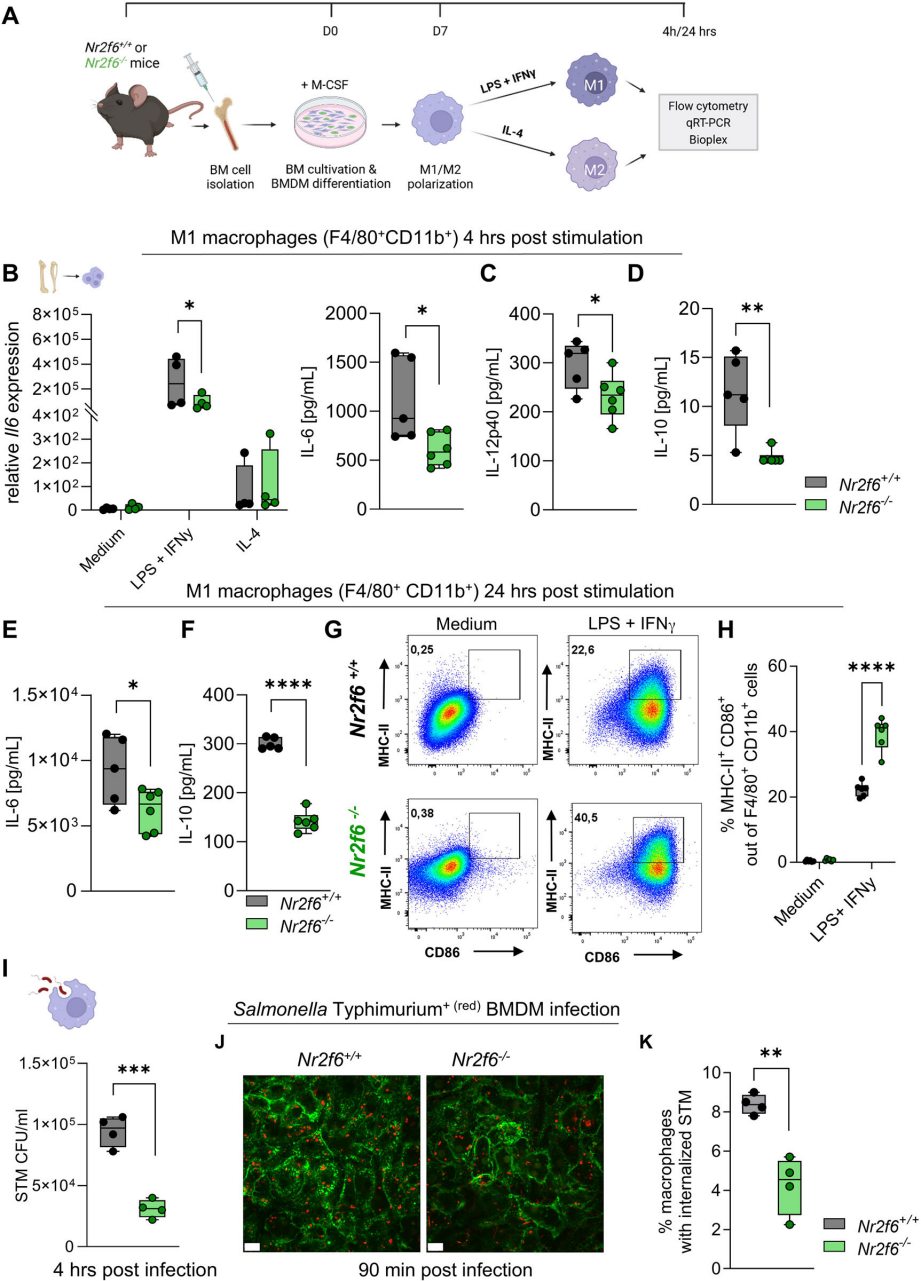
Intrinsic loss of NR2F6 in BMDMs alters M1 responses and *Salmonella* Typhimurium phagocytosis in vitro. A) Scheme depicting the experimental procedure of BMDM differentiation and polarization. Bone marrow cells were cultured with M‐CSF for 7 days and treated with LPS + IFNγ or IL‐4 for 4 or 24 h before harvest and analysis. B) Quantification of *Il6* expression and IL‐6, C) IL‐12p40, and D) IL‐10 cytokine secretion into the supernatant of 4 h activated M1 (LPS+IFNγ) or M2 (IL‐4) wild‐type (*Nr2f6^+/+^*) or *Nr2f6*‐deficient BMDMs. E) Quantification of IL‐6 and F) IL‐10 cytokine secretion into the supernatant of 24 h activated M1 wild‐type (*Nr2f6^+/+^*) or *Nr2f6*‐deficient (*Nr2f6^−/−^*) BMDMs. G) Representative dot‐plots and H) quantification of frequencies of activated MHC‐II^+^CD86^+^ macrophages of 24 h activated M1 wild‐type (*Nr2f6^+/+^*) or *Nr2f6*‐deficient (*Nr2f6^−/−^*) BMDMs. I) Quantification of colony‐forming units (CFUs) from wild‐type (*Nr2f6^+/+^*) or *Nr2f6*‐deficient (*Nr2f6^−/−^*) *Salmonella* Typhimurium infected BMDMs. J) Representative images of *Salmonella* Typhimurium (RFP) infected BMDMs stained with wheat germ agglutinin, scale bars, 13 µm. K) Flow cytometric quantification of fluorescent intracellular *Salmonella* Typhimurium burden in wild‐type (*Nr2f6^+/+^*) or *Nr2f6*‐deficient (*Nr2f6^−/−^*) BMDMs 90 min. post‐infection. Representative data shown are from at least two independent experiments with *n* = 2‐3 per group and experiment. Each dot represents the data of an individual mouse‐derived BMDM culture. Results are shown as median ± IQR with whiskers from min. to max. The Shapiro‐Wilk test evaluated the normality of data. Asterisks indicate statistically significant differences between genotypes calculated using the Student's *t*‐test or Mann‐Whitney *U* test for non‐parametric data. A *p*‐value < 0.05 was considered statistically significant, *0.05,**0.01, ***0.001, ****0.0001 (See also Figure S3, Supporting Information).

Discrepant findings regarding the influence of NR2F6 on cytokine regulation in the human cell line HEK 293T and mouse bone marrow‐derived macrophages (BMDMs) have been reported.^[^ ^36^, ^37^ ^]^ To investigate the cell‐intrinsic roles of NR2F6 in macrophages, we differentiated wild‐type or *Nr2f6*‐deficient BMDMs over 7 days. Subsequently, we polarized them with M1 (LPS+IFNγ) or M2 (IL‐4) activating conditions (**Figure** **3**A; Figure S3A Supporting Information). In vitro BMDM differentiation, as confirmed through CD11b^+^F4/80^+^ and viability staining, was comparable between genotypes (Figure S3B Supporting Information). Following stimulation, key M1 and M2 markers, such as inducible nitric oxide synthase (*iNOS*) (M1) or arginase1 (*Arg1*) (M2), were robustly induced in both genotypes, with a minor reduction in *Arg1* expression in *Nr2f6*‐deficient BMDMs following IL‐4 stimulation (Figure S3C,D Supporting Information).

After 4 h of M1 polarization, *Nr2f6*‐deficient BMDMs exhibited a significant reduction in both mRNA expression and secretion of the pro‐inflammatory cytokine IL‐6 compared to wild‐type controls (Figure 3B). Additionally, IL‐12p40 and IL‐10 secretion levels were lower in *Nr2f6*‐deficient M1 polarized BMDMs (Figure 3C,D). This trend persisted 24 h after M1 stimulation conditions, as reflected by significantly reduced IL‐6 and particularly IL‐10 secretion in *Nr2f6*‐deficient M1 BMDMs (Figure 3E,F). Flow cytometric analysis revealed a substantial increase in the number of the activation marker major histocompatibility complex‐II (MHC‐II) expressing cells within CD11b^+^F4/80^+^ cells in *Nr2f6*‐deficient M1 BMDMs, enhancing the percentage of activated CD86^+^MHC‐II^+^ double‐positive *Nr2f6*‐deficient M1 BMDMs population compared to wild‐type controls (Figure 3G,H; Figure S3E,F Supporting Information).

As a next step, we wondered if the anti‐bacterial properties of *Nr2f6*‐deficient BMDMs were altered, therefore, we exposed BMDMs to *Salmonella* Typhimurium in vitro. Bacterial colony forming units (CFUs) were significantly reduced in cultures of *Nr2f6*‐deficient BMDMs already after 30 min but also after 4 h compared to wild‐type controls at both time points (Figure 3I; Figure S3G Supporting Information). Confocal microscopy and flow cytometric analysis, using an RFP‐labeled *Salmonella* Typhimurium strain, independently confirmed this observation by showing a reduction in the relative number of *Nr2f6*‐deficient BMDMs infected with *Salmonella* Typhimurium (Figure 3J,K). Notably, since *Salmonella* Typhimurium can enter macrophages through both active invasion and classical phagocytosis, our internalization assays reflect the combined contribution of these two mechanisms.

Therefore, the intrinsic absence of NR2F6 alters M1 BMDM responses, characterized by reduced secretion of both pro‐ and anti‐inflammatory cytokines, elevated frequencies of activated MHC‐II^+^ cells, and decreased incorporation of *Salmonella* Typhimurium. These results suggest that the absence of NR2F6 interferes with initial events during phagocytosis of *Salmonella* Typhimurium into host BMDMs in vitro.

### 2.4 Loss of NR2F6 Protects Mice During *Salmonella* Typhimurium Infection

**Figure 4:**
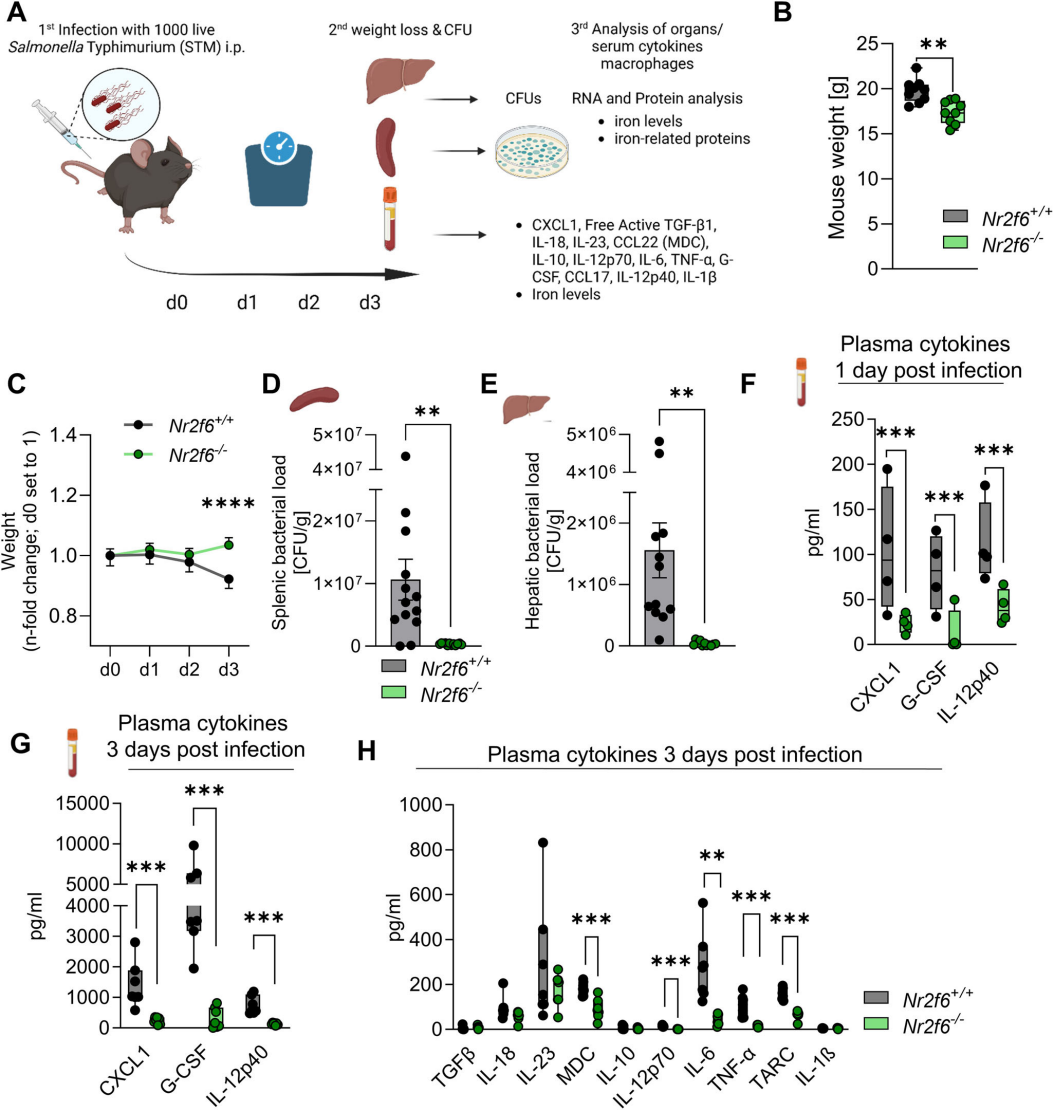
Loss of NR2F6 protects mice from *Salmonella* Typhimurium infection. A) Scheme of experimental setup following *Salmonella* Typhimurium infection. B) Baseline weight and C) relative weight loss over the course of *Salmonella* Typhimurium infection of wild‐type (*Nr2f6^+/+^*) or *Nr2f6*‐deficient (*Nr2f6^−/−^*) mice. D) Splenic and E) liver colony forming units (CFU) per gram of wild‐type (*Nr2f6^+/+^*) or *Nr2f6*‐deficient (*Nr2f6^−/−^*) organ 72 h post‐*Salmonella* Typhimurium infection. F) Plasma cytokine levels of wild‐type (*Nr2f6^+/+^*) or *Nr2f6*‐deficient (*Nr2f6^−/−^*) mice 22 h post *Salmonella* Typhimurium infection. G,H) Plasma cytokine and chemokine levels of wild‐type (*Nr2f6^+/+^*) or *Nr2f6*‐deficient (*Nr2f6^−/−^*) mice 72 h post *Salmonella* Typhimurium infection. Representative data shown are from at least two (for plasma cytokines 22 h one) independent experiments with *n* = 4 per group and experiment, total *n* = 10/10, and *n* = 4/4 (for plasma cytokines 22 h) (*Nr2f6^+/+^*)/(*Nr2f6^‐/‐^*). Each dot represents the data from one individual mouse. Results are shown as median ± IQR with whiskers from min. to max (B,F–H) or mean ± SEM. The Shapiro‐Wilk test evaluated the normality of data. Asterisks indicate statistically significant differences between genotypes calculated using the Student's *t*‐test or Mann‐Whitney *U* test for non‐parametric data. A *p*‐value < 0.05 was considered statistically significant, **0.01, ***0.001, ****0.0001 (See also Figure S4, Supporting Information).

To define the functional importance of NR2F6 within macrophages during bacterial pathogen encounters in vivo, we infected *Nr2f6*‐deficient and wild‐type control mice intraperitoneally with *Salmonella* Typhimurium, a siderophilic intracellular gram‐negative bacterium. We assessed weight loss, bacterial burden (CFUs), transcriptional levels, and abundance of iron‐metabolism proteins within the spleen and liver 72 h post‐infection and quantified plasma cytokine levels after 22 and 72 h (**Figure** **4**A). Notably, the baseline weight of *Nr2f6*‐deficient mice was lower in animals aged between 8 to 12 weeks (Figure 4B). Despite this, *Nr2f6*‐deficient mice were protected against weight loss induced by bacterial infection (Figure 4C; Figure S4A Supporting Information). Three days post‐*Salmonella* Typhimurium infection, we observed a substantial reduction in bacterial CFUs within the spleens and livers of *Nr2f6*‐deficient mice compared to wild‐type (Figure 4D,E). Turning our attention to plasma cytokines, we identified significant reductions in chemokine (C‐X‐C motif) ligand 1 (CXCL1), granulocyte colony‐stimulating factor (G‐CSF), and IL‐12p40 levels in *Nr2f6*‐deficient mouse plasma one‐day post‐infection compared to infected wild‐type controls (Figure 4F; Figure S4B Supporting Information). Although these cytokine levels increased until day 3, they remained markedly lower in *Nr2f6*‐deficient plasma than in wild‐type controls (Figure 4G). Additionally, macrophage‐derived chemokine (MDC), IL‐12p70, IL‐6, tumor necrosis factor (TNF)α, and thymus and activation‐regulated chemokine (TARC) levels were also lower in the plasma of *Nr2f6*‐deficient mice three days post‐*Salmonella* Typhimurium infection when compared to wild‐type (Figure 4H). No differences in the plasma levels of transforming growth factor (TGF)β, IL‐18, IL‐10, or IL‐1β were observed (Figure 4H). Importantly, when examining cytokine levels within the plasma of healthy mice, no notable differences were observed between the genotypes, except for heightened IL‐23 levels (Figure S4C Supporting Information).

Collectively, our dataset, for the first time, provides compelling evidence of an indispensable regulatory role for NR2F6 during *Salmonella* Typhimurium infection. The loss of NR2F6 protected mice against infection‐driven weight loss, coinciding with reduced bacterial burden and subsequently strongly reduced pro‐inflammatory plasma cytokine levels.

### 2.5 Host Iron Homeostasis is Altered in *Nr2f6*‐deficient Mice Following *Salmonella* Typhimurium Infection

*Salmonella* Typhimurium‐induced septicemia causes hypoferremia by upregulation of IL‐6 mediated induction of the nuclear hormone receptor ERRγ and the subsequent induction of the iron regulatory hormone hepcidin in the liver.^[^ ^35^, ^47^, ^48^ ^]^ To explore iron homeostasis in *Nr2f6*‐deficient mice, we analyzed iron levels and the expression of essential iron‐metabolism proteins involved in the uptake, storage, and export of iron, namely the transferrin receptor protein 1 (encoded by *Trfc*/*Tfr1*), ferritin heavy chain (encoded by *Fth1*), and ferroportin (encoded by *Slc40a1*/*Fpn1*) of healthy and *Salmonella* Typhimurium infected mice.

**Figure 5:**
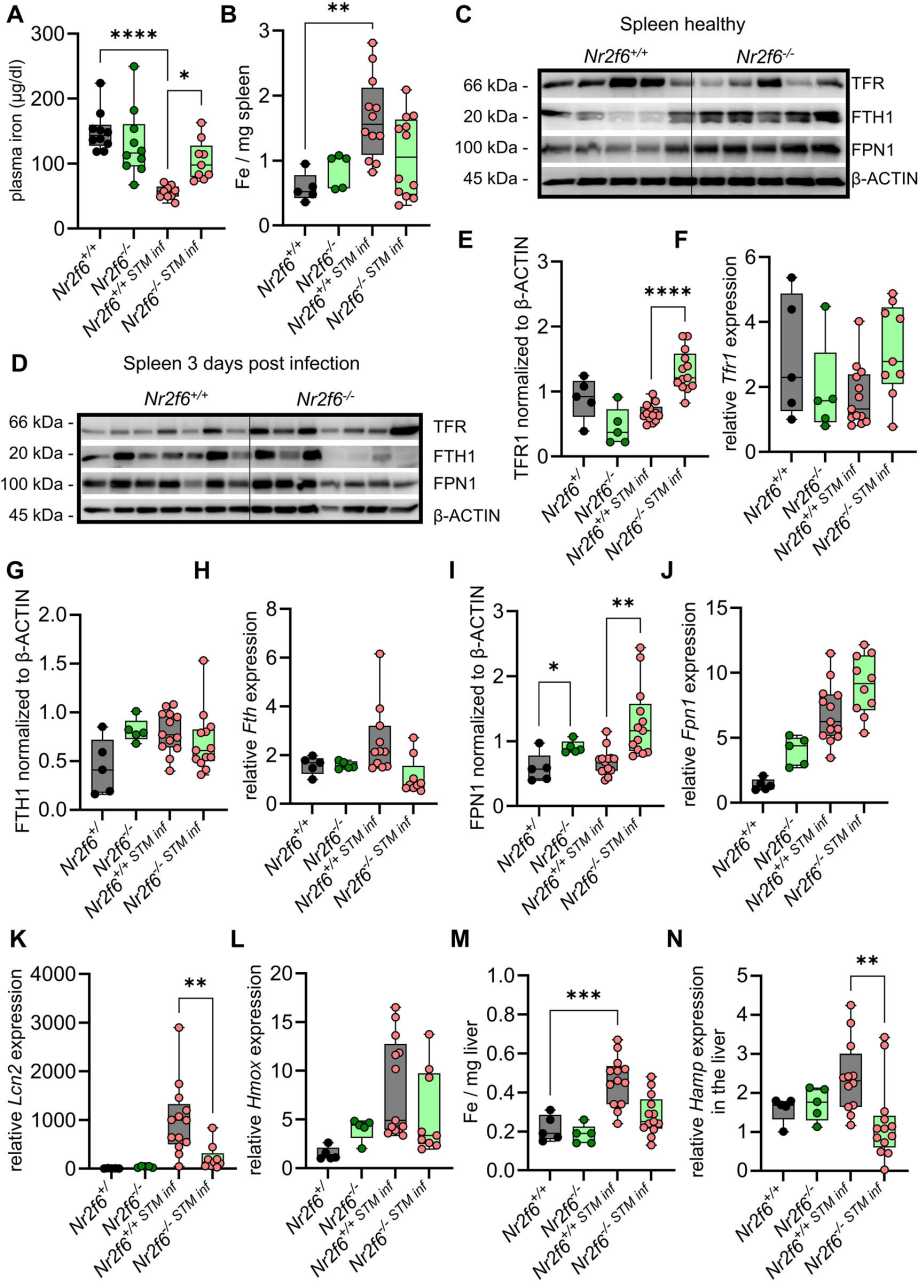
Loss of NR2F6 alters iron‐related proteins in the spleen, particularly after *Salmonella* infection. A) Plasma iron levels of healthy wild‐type (*Nr2f6^+/+^*) or *Nr2f6*‐deficient (*Nr2f6^−/−^*) mice and 72 h post *Salmonella* Typhimurium infection. B) Quantification of iron (Fe) levels per mg spleen of healthy wild‐type (*Nr2f6^+/+^*) or *Nr2f6*‐deficient (*Nr2f6^−/−^*) mice. C) Representative protein abundance of the transferrin receptor1 (TFR1), ferritin (FTH1), ferroportin1 (FPN1), and β‐ACTIN in the spleen of healthy, or D) 72 h post *Salmonella* Typhimurium infection of wild‐type (*Nr2f6^+/+^*) or *Nr2f6*‐deficient (*Nr2f6^−/−^*) mice. E) Representative protein abundance of TFR1 normalized to β‐ACTIN. F) Quantification of fold change *Tfr1* mRNA expression relative to *Hprt* in the spleen of healthy or 72 h post *Salmonella* Typhimurium infection of wild‐type (*Nr2f6^+/+^*) or *Nr2f6*‐deficient (*Nr2f6^−/−^*) mice. G) Representative protein abundance of FTH1 normalized to β‐ACTIN. H) Quantification of fold change *Fth* mRNA expression relative to *Hprt* in the spleen of healthy or 72 h post *Salmonella* Typhimurium infection of wild‐type (*Nr2f6^+/+^*) or *Nr2f6*‐deficient (*Nr2f6^−/−^*) mice. I) Representative protein abundance of FPN1 normalized to β‐ACTIN. J) Quantification of fold change *Fpn1* mRNA expression relative to *Hprt* in the spleen of healthy or 72 h post *Salmonella* Typhimurium infection of wild‐type (*Nr2f6^+/+^*) or *Nr2f6*‐deficient (*Nr2f6^−/−^*) mice. K) Quantification of fold change *Lcn2* and L) *Hmox* mRNA expression relative to *Hprt* in the spleen of healthy or 72 h post *Salmonella* Typhimurium infection of wild‐type (*Nr2f6^+/+^*) or *Nr2f6*‐deficient (*Nr2f6^−/−^*) mice. M) Quantification of iron (Fe) levels per mg liver of healthy wild‐type (*Nr2f6^+/+^*) or *Nr2f6*‐deficient (*Nr2f6^−/−^*) mice. N) Quantification of fold change *Hamp* mRNA expression relative to *Hprt* in the spleen of healthy or 72 h post *Salmonella* Typhimurium infection of wild‐type (*Nr2f6^+/+^*) or *Nr2f6*‐deficient (*Nr2f6^−/−^*) mice. Representative data shown are from at least two independent experiments with *n* = 2‐3 per group and experiment, total *n* = 13/9, and *n* = 5/5 (for steady state) (*Nr2f6^+/+^*)/(*Nr2f6^‐/‐^*). Each dot represents the data from one individual mouse. Quantification of mRNA fold change was calculated relative to one uninfected wild‐type (*Nr2f6^+/+^*) set as 1. Results are shown as median ± IQR with whiskers from min. to max. The Shapiro‐Wilk test evaluated the normality of data. Asterisks indicate statistically significant differences between genotypes calculated using the one‐way ANOVA. A *p*‐value < 0.05 was considered statistically significant, **0.01, ***0.001, ****0.0001 (See also Figure S5, Supporting Information).

We did not detect differences in iron levels in the plasma and spleen in healthy animals between the genotypes (**Figure** **5**A,B). Following *Salmonella* Typhimurium infection, plasma and splenic iron levels in *Nr2f6*‐deficient mice were not changed compared to healthy *Nr2f6*‐deficient controls. In contrast, in wild‐type mice, plasma iron levels were significantly reduced upon infection, but splenic and liver iron levels were enhanced (Figure 5A,B), the latter in line with the well‐described metabolic iron response to systemic infection.^[^ ^10^ ^]^

In the spleen of healthy mice, the TFR1 protein and fold induction of *Tfr1* gene expression, the latter normalized to wild‐type controls, were comparable. However, during *Salmonella* Typhimurium infection, TFR1 protein levels were elevated in spleens of *Nr2f6*‐deficient compared to infected wild‐type mice, being in line with a tendency for lower splenic iron levels (Figure 5C–F). FTH1 protein and fold induction of *Fth1* gene expression in the spleen of healthy or infected *Nr2f6*‐deficient mice was comparable to wild‐type controls (Figure 5C,D,G,H). FPN1 protein and fold induction of *Fpn1* gene expression levels were elevated in both healthy and infected *Nr2f6*‐deficient mice, but only protein levels were enhanced compared to wild‐type controls, with a significant increase of the expression levels following *Salmonella* Typhimurium infection (Figure 5C,D,I,J). Corresponding to the reduced cytokine levels in the plasma of *Nr2f6*‐deficient mice, fold induction of the inflammation marker lipocalin‐2 (*Lcn2*) was significantly lower in the spleens of infected *Nr2f6*‐deficient mice compared to wild‐type controls, whereas fold induction of heme oxygenase (*Hmox*) gene expression was not altered between the genotypes (Figure 5K,L).

*Salmonella* Typhimurium infection significantly enhanced hepatic iron in wild‐type but not in *Nr2f6*‐deficient mice (Figure 5M). Consistent with this finding, hepcidin (*Hamp*) expression in the liver of *Nr2f6*‐deficient infected mice was significantly reduced compared to wild‐type, while *Hamp* levels were unchanged between genotypes in steady‐state (Figure 5N).

In healthy livers, iron levels, protein, and fold induction of TFR1, FTH1, and FPN1 gene expression were similar in both genotypes (Figure 5M; Figure S5A–H Supporting Information). Following infection with *Salmonella* Typhimurium ‐ and mirroring the splenic results ‐ relative TFR1 protein and fold induction of *Tfr1* gene expression were significantly increased in the liver of *Nr2f6*‐deficient mice (Figure S5A–D Supporting Information). In contrast, FTH1 protein levels were reduced in the liver of *Nr2f6*‐deficient mice, whereas FPN1 protein levels were enhanced (Figure S25B and S5E–H Supporting Information). In parallel to the spleen *Lcn2, Hmox* expression in the liver of infected *Nr2f6‐*deficient mice was decreased compared to infected wild‐type controls (Figure S5I,J Supporting Information).

These findings suggest that iron metabolism in the steady state in the blood, spleen, and liver is not altered between the genotypes. In contrast, *Salmonella* Typhimurium infection leads to substantial changes in iron metabolism in wild‐type mice, whereas plasma, splenic, and liver iron levels in *Nr2f6*‐deficient mice were comparable to uninfected *Nr2f6*‐deficient controls. In accordance with the reduced hepcidin levels in the liver of infected *Nr2f6*‐deficient mice, FPN1 and TFR1 protein levels in both the spleen and liver were enhanced.

### 2.6 Altered Transcriptional Landscape in *Nr2f6‐*deficient RPMs

**Figure 6:**
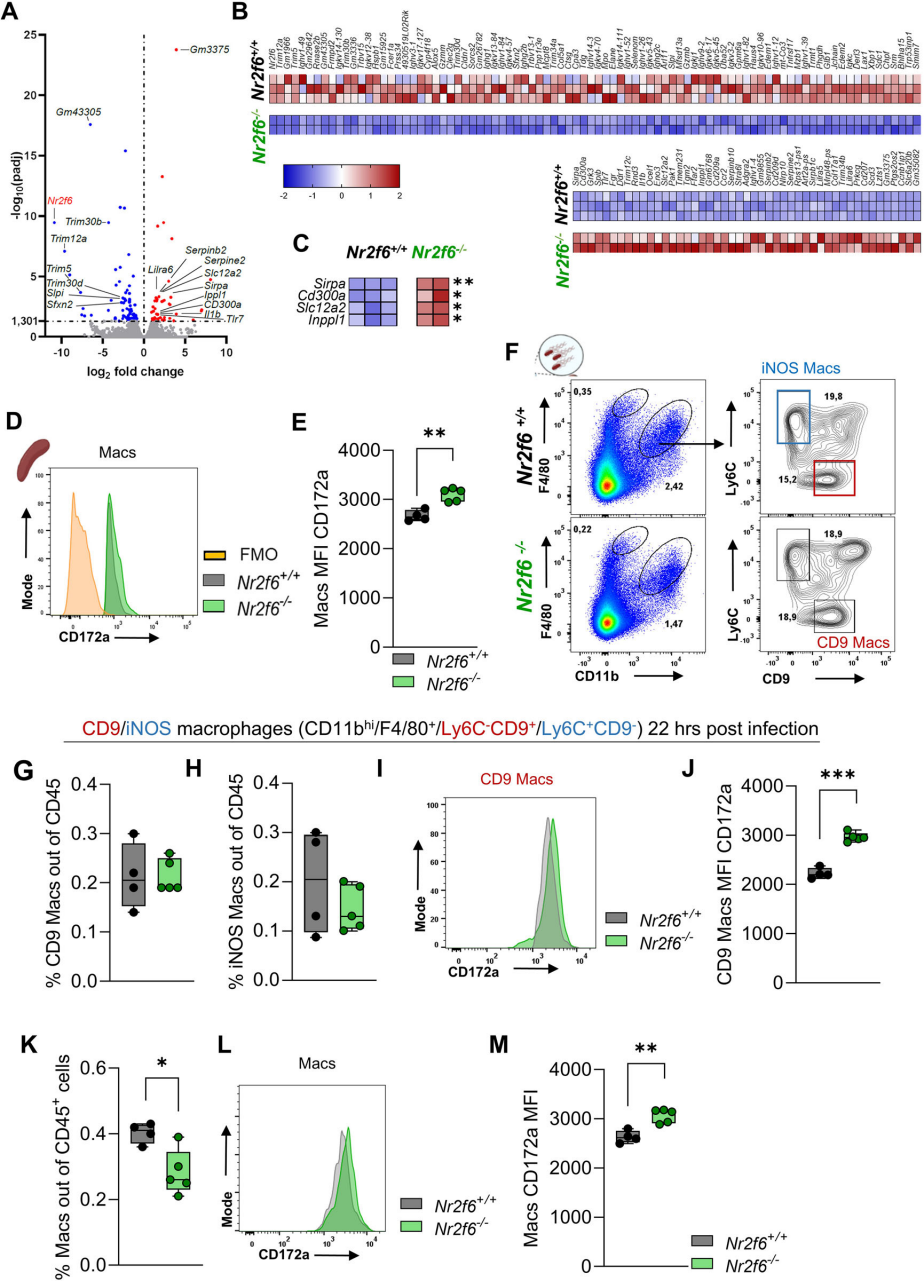
Loss of NR2F6 alters the expression of genes involved in phagocytosis in RPMs. A) Volcano plot representation of differentially expressed genes (DEGs) between wild‐type (*Nr2f6^+/+^*) or *Nr2f6*‐deficient (*Nr2f6^−/−^*) RPMs. DEG are selected by DESeq2 (adjusted p‐value (padj)< 0.05). B) Heatmap of DEG between wild‐type (*Nr2f6^+/+^*) or *Nr2f6*‐deficient RPMs were defined by adjusted p‐value (padj) < 0.05 and z‐score normalized. C) Heatmap of DEG involved in phagocytic responses. A padj‐value < 0.05 was considered statistically significant. * *padj* < 0.05,** *padj* < 0.01. D) Histogram and E) quantification of Sirpα (CD172a) MFI of wild‐type (*Nr2f6^+/+^*) or *Nr2f6*‐deficient splenic macrophages. F) Representative dot‐plots of CD11b^int^/F4/80^+^ splenic macrophages, iNOS, and CD9^+^ macrophages out of splenic CD11b^+^/F4/80^int^ cells of wild‐type (*Nr2f6^+/+^*) or *Nr2f6*‐deficient (*Nr2f6^−/−^*) *Salmonella* Typhimurium infected mice after 22 h. G) Quantification of frequencies of splenic CD9^+^ and H) iNOS macrophages out of CD45^+^ cells of wild‐type (*Nr2f6^+/+^*) or *Nr2f6*‐deficient (*Nr2f6^−/−^*) mice. I) Histogram and J) quantification of Sirpα (CD172a) MFI of wild‐type (*Nr2f6^+/+^*) or *Nr2f6*‐deficient (*Nr2f6^−/−^*) splenic CD9^+^ macrophages 22 h post‐infection. K) Quantification of frequencies of CD11b^int^/F4/80^+^ splenic macrophages out of CD45^+^ cells of wild‐type (*Nr2f6^+/+^*) or *Nr2f6*‐deficient mice. L) Histogram and M) quantification of Sirpα (CD172a) MFI of wild‐type (*Nr2f6^+/+^*) or *Nr2f6*‐deficient splenic macrophages 22 h post‐infection. Data shown are from one RNA‐Seq sort experiment with *n* = 3/2 (*Nr2f6^+/+^*)/(*Nr2f6^‐/‐^*); flow cytometric data was generated from at least two experiments, with *n* = 2‐3 per group and experiment, total *n* = 4/5 (for CD172a) under healthy conditions and following 22 h after *Salmonella* Typhimurium infection (*Nr2f6^+/+^*)/(*Nr2f6^‐/‐^*). Each dot represents the data from one individual mouse. Results are shown as median ± IQR with whiskers from min. to max. The Shapiro‐Wilk test evaluated the normality of data. Asterisks indicate statistically significant differences between genotypes calculated using the Student's *t*‐test or Mann‐Whitney *U* test for non‐parametric data. A *p*‐value < 0.05 was considered statistically significant. *0.05,**0.01, ***0.001 (See also Figure S6, Supporting Information).

We employed an unbiased approach to explore the transcriptional landscape of NR2F6‐regulated genes in splenic RPMs to reveal underlying mechanisms. We FACS sorted Lin^‐^F4/80^hi^CD11b^int^CD106^+^CD64^‐^CD169^‐^ RPMs from wild‐type and *Nr2f6*‐deficient spleens and performed bulk RNA sequencing. (Figure S6A Supporting Information). Subsequently, we identified 135 differentially expressed genes (DEGs) in *Nr2f6*‐deficient RPMs (**Figure** **6**A,B). Of these genes, 47 exhibited upregulation in *Nr2f6*‐deficient RPMs, whereas 88 genes displayed downregulation compared to wild‐type controls (Figure 6B).

Given that NR2F6 predominantly functions as a transcriptional repressor, we focused on genes with heightened expression in *Nr2f6*‐deficient RPMs. Enhanced transcription rates were observed in the expression of Leukocyte immunoglobulin‐like receptor, subfamily A (with TM domain), member 6 (*Lilra6*); serine (or cysteine) peptidase inhibitor, clade E, member 2 (*Serpin2*), (*Serpinb2*), inositol polyphosphate phosphatase‐like 1 (*Inppl1*; SHIP‐2 protein); solute carrier family 12, member 2 (*Slc12a2*; NKCC1 protein); interleukin 1 beta (*Il1b*); toll‐like receptor 7 (*Tlr7*); CD300A molecule (*Cd300a*) and signal‐regulatory protein alpha (*Sirpa, Cd172a*) in *Nr2f6*‐deficient RPMs. (Figure 6A–C). Notably, three of these receptors, namely Sirpα, Slc12a2, and CD300a, and the signaling molecule SHIP‐2 are involved in phagocytic responses, with two receptors being crucial for transmitting “don't‐eat‐me” signals (Figure 6C).^[^ ^49^, ^50^, ^51^, ^52^, ^53^, ^54^ ^]^ Of note, the engagement of Sirpα by CD47 not only regulates phagocytosis but also prevents phagocyte migration and activation.^[^ ^55^, ^56^, ^57^ ^]^

Simultaneously, we investigated downregulated genes. As expected, *Nr2f6* expression was substantially reduced, in addition, the expression of family members of the Trim family (*Trim 12a*, *Trim5*, and *Trim 30b&d*); secretory leukocyte peptidase inhibitor (*Slpi*); sideroflexin 2 (*Sfxn2*); and the protein phosphatase 1 or regulatory subunit 3 (*Ppp1r3e*) were downregulated in *Nr2f6*‐deficient RPMs (Figure 6A,B). Loss of NR2F6 did not alter the expression of RPM‐related signature genes, including *Bach1, Pparg, Spic, or Treml4*.^[^ ^33^, ^58^ ^]^

Considering the outcomes from our RNAseq dataset, we chose the “don't‐eat‐me” checkpoint Sirpα (CD172a) for further validation, especially as Sirpα (CD172a) expression levels (MFI) were enhanced in splenic macrophages of *Nr2f6*‐deficient mice (Figure 6D,E).

*Salmonella* Typhimurium infection follows eclipse‐like dynamics with a first phase of bacterial control mediated by tissue‐resident red‐pulp macrophages, whereas the second phase is dependent on CD9^+^ macrophages originating from non‐classical monocytes (NCM).^[^ ^21^ ^]^ It has been reported that depletion of CD9^+^ macrophages reduces *Salmonella* Typhimurium infection and prolongs mice survival.^[^ ^21^ ^]^ Therefore, we investigated the frequencies of *Nr2f6*‐deficient splenic F4/80^hi^CD11b^int^ (Macs), CD9^+^, and iNOS expressing macrophages, focusing on Sirpα expression 22 h post‐infection.

During this early phase of *Salmonella* Typhimurium infection, the frequencies of iNOS^+^ (Ly6C^+^CD9^‐^) and CD9^+^ (Ly6C^‐^CD9^+^) macrophages within the splenic CD45^+^ cells remained comparable between *Nr2f6*‐deficient and wild‐type mice, while the frequency of splenic macrophages was already reduced compared to healthy controls and still lower in *Nr2f6*‐deficient mice when compared to infected wild‐type controls (Figure 6F–K).

In iNOS^+^ macrophages, the MFI of Sirpα was unaltered in *Nr2f6*‐deficient mice, whereas in CD9^+^ and F4/80^hi^CD11b^int^ (Macs) subsets, the MFI of Sirpα was significantly increased when compared to infected wild‐type controls (Figure 6I,J,L,M; Figure S6B Supporting Information).

Taken together, RNAseq analysis of *Nr2f6*‐deficient RPMs revealed an altered transcriptional landscape with upregulated expression of genes involved in phagocytosis, such as Sirpα. Along this line an enhanced MFI of Sirpα could be detected in healthy splenic macrophages as well as both splenic and CD9^+^ macrophages during the early course of *Salmonella* Typhimurium infection of *Nr2f6*‐deficient mice.

### 2.7 Lower Phagocytic Potential in *Nr2f6*‐deficient Macrophages In Vitro

**Figure 7:**
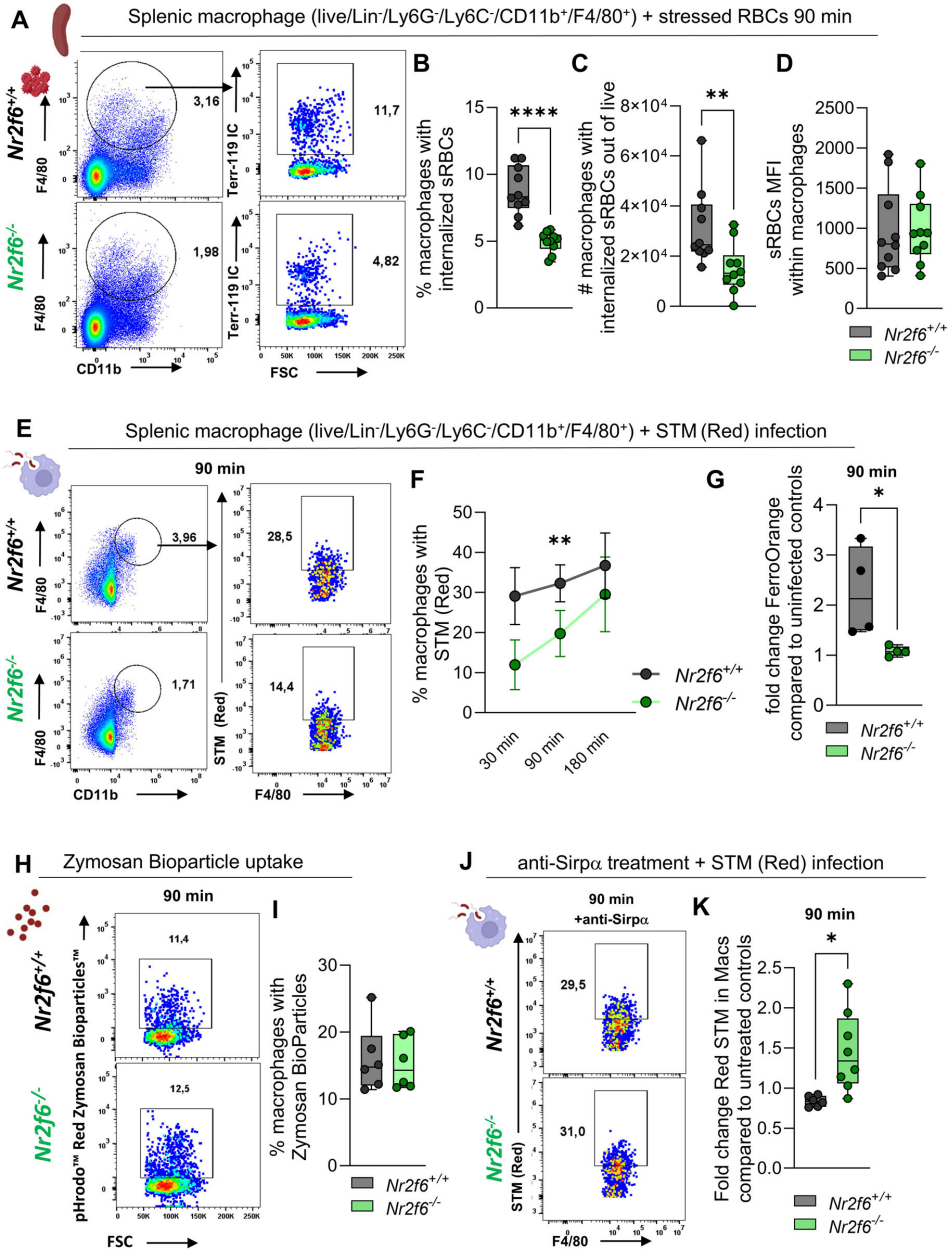
Reduced phagocytosis in splenic *Nr2f6*‐deficient macrophages in vitro. A) Representative dot‐plots and quantification of B) frequencies, C) total cell numbers, and D) MFI of stressed red blood cell (sRBC) phagocytosis into macrophages of wild‐type (*Nr2f6^+/+^*) or *Nr2f6*‐deficient (*Nr2f6^−/−^*) spleens. E) Representative dot‐plots and quantification of F) frequencies of *Salmonella* Typhimurium infection into macrophages after 30, 90, and 180 min. of wild‐type (*Nr2f6^+/+^*) or *Nr2f6*‐deficient (*Nr2f6^−/−^*) spleens. G) Relative quantification of intracellular ferrous (Fe^2+^) iron with FerroOrange in total splenocytes 90 min after *Salmonella* Typhimurium infection. H) Representative dot‐plots and quantification of I) frequencies of Zymosan BioParticle phagocytosis into macrophages of wild‐type (*Nr2f6^+/+^*) or *Nr2f6*‐deficient (*Nr2f6^−/−^*) spleens. J) Representative dot‐plots and K) relative quantification of *Salmonella* Typhimurium infection after 30 min of anti‐Sirpα antibody pre‐incubation and 90 min post‐infection into macrophages of wild‐type (*Nr2f6^+/+^*) or *Nr2f6*‐deficient (*Nr2f6^−/−^*) spleens. Representative data shown are from at least two independent experiments with *n* = 4 per group and experiment. Each dot represents the data from one individual mouse. Results are shown as mean ± SEM (F) median ± IQR with whiskers from min. to max. The Shapiro‐Wilk test evaluated the normality of data. Asterisks indicate statistically significant differences between genotypes calculated using the Student's *t*‐test or Mann‐Whitney *U* test for non‐parametric data. A *p*‐value < 0.05 was considered statistically significant. *0.05,**0.01, ***0.001, ****0.0001 (See also Figure S7, Supporting Information).

To assess the phagocytic capacity of *Nr2f6*‐deficient splenic macrophages under steady‐state conditions in a Sirpα‐CD47 dependent way, we set up an RBC phagocytosis assay following Akilesh's protocol.^[^ ^59^ ^]^ Heat‐induced stressed RBCs (sRBC) were incubated with wild‐type or *Nr2f6^‐/‐^* splenocytes at a 10:1 ratio for 90 min. Following the lysis of non‐engulfed sRBCs, the percentage of phagocytosed sRBCs within splenic macrophages was quantified by flow cytometric analysis of intracellular Ter119 (Figure S7A–C Supporting Information). The frequency and total cell counts, though not the MFI, of *Nr2f6*‐deficient macrophages that phagocytosed stressed RBCs were significantly reduced (**Figure** **7**A–D).

Expanding our analysis to pathogen uptake, we utilized a fluorescent *Salmonella* Typhimurium (RFP^+^) strain and investigated internalization into splenic macrophages. The numbers of *Nr2f6*‐deficient macrophages that internalized *Salmonella* Typhimurium (RFP^+^) in vitro were substantially reduced, while the internalization per cell (MFI) was unchanged (Figure 7E; Figure S7D Supporting Information). To identify underlying mechanisms, we investigated the time kinetic of *Salmonella* Typhimurium (RFP^+^) uptake after 30, 90, and 180 min of infection initiation. The percentages and total cell numbers of *Nr2f6*‐deficient macrophages that internalized *Salmonella* Typhimurium (RFP^+^) were low after 30 min but increased over time when compared to wild‐type controls (Figure 7F; Figure S7E Supporting Information). Our internalization assays reflect the combined contribution of active invasion and phagocytosis.

In parallel, we investigated the free intracellular ferrous (Fe^2+^) iron ion content via FerroOrange determination, which was significantly reduced in infected *Nr2f6*‐deficient splenocytes when compared to wild‐type (Figure 7G).

Expanding our analysis to a broader scale and to investigate pathogen phagocytosis independent of Sirpα, we utilized pHrodo^TM^ red BioParticles containing eukaryote *Saccharomyces cerevisiae* (Zymosan) conjugate, which only fluoresces following intracellular uptake and transport into the phagolysosome, being dependent on Dectin‐1 or the mannose receptor for phagocytosis. Phagocytosis of Zymosan bead conjugate was comparable between the genotypes, indicating that phagocytosis is not generally comprised in *Nr2f6*‐deficient macrophages (Figure 7H,I).

Considering the literature on genes regulating phagocytosis, such as Sirpα, SLC12A2 (NKCC1), or CD330a – which are altered in *Nr2f6*‐deficient RPMs ‐ and based on our results obtained thus far ‐ we focused on the functional roles of the Sirpα axis during phagocytosis. To establish a functional relationship of Sirpα  binding with phagocytic activity, we preincubated splenocytes of both genotypes with anti‐Sirpα antibody followed by 90 min of *Salmonella* Typhimurium (RFP^+^) infection. In our experimental setting, 30 min of anti‐Sirpα pre‐treatment did not alter the phagocytic activity of wild‐type macrophages but enhanced the phagocytic incorporation of *Salmonella* Typhimurium (RFP^+^) into *Nr2f6*‐deficient macrophages, thereby leading to a significantly increased fold change when compared to the induction in wild‐type cells (Figure 7J,K; Figure S7F Supporting Information).

Taken together, the loss of NR2F6 resulted in reduced phagocytic responses of macrophages to stressed RBCs and *Salmonella* Typhimurium. Pre‐incubation with anti‐Sirpα partially enhanced the phagocytic activity of *Nr2f6*‐deficient macrophages for *Salmonella* Typhimurium.

### 2.8 Anti‐Sirpα Treatment Alters Bacterial Responses in *Nr2f6*‐deficient Mice during Infection in vivo

To investigate the role of Sirpα in *Nr2f6*‐deficient mice, we administered an anti‐Sirpα blocking antibody (P84) via intraperitoneal injection on days ‐1, 1, and 2 of *Salmonella* Typhimurium infection. Weight loss and bacterial growth in the spleen and liver were assessed 72 h post‐infection. We selected the allosteric Sirpα ‐ antibody (P84), which selectively blocks Sirpα signaling without disrupting CD47 interaction, instead of the MY1 antibody that inhibits both Sirpα and Sirpβ and blocks CD47 binding.^[^ ^60^, ^61^, ^62^ ^]^

**Figure 8:**
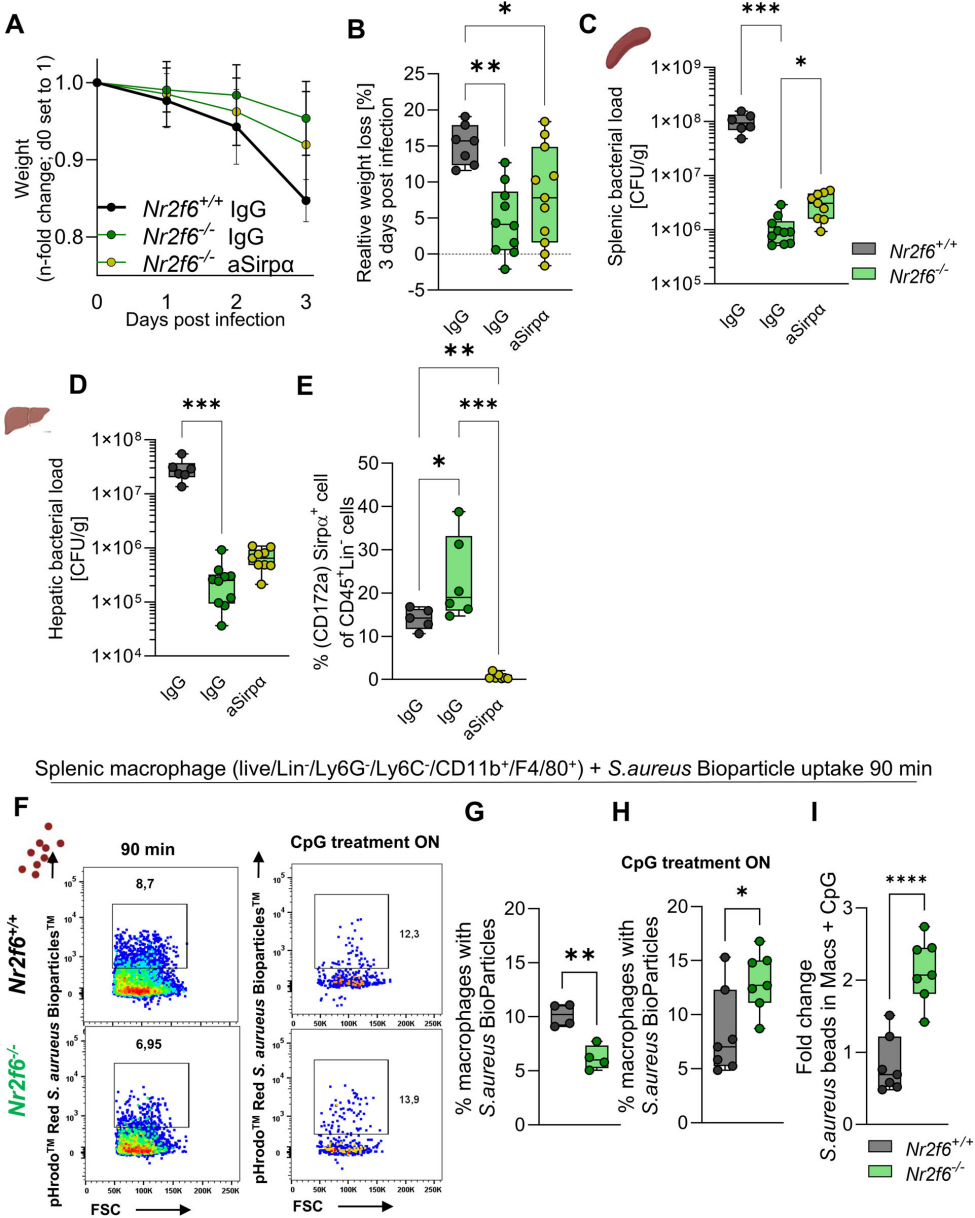
Sirpα blocking partially adjusts *Nr2f6*‐deficient macrophage phagocytic uptake of *Salmonella* Typhimurium to wild‐type in vivo. A) Baseline weight over the course of *Salmonella* Typhimurium infection and B) relative weight loss of IgG treated (d‐1, d1, d2) wild‐type (*Nr2f6^+/+^*) or *Nr2f6*‐deficient (*Nr2f6^−/−^*) mice and anti‐Sirpα treated *Nr2f6*‐deficient (*Nr2f6^−/−^*) mice. C) Splenic and D) liver colony forming units (CFU) per gram of wild‐type (*Nr2f6^+/+^*) or *Nr2f6*‐deficient (*Nr2f6^−/−^*) organ 72 h post‐*Salmonella* Typhimurium infection. E) Quantification of CD172a^+^ cells out of CD45^+^ Lin^‐^cells in the blood of IgG treated (d‐1, d1, d2) wild‐type (*Nr2f6^+/+^*) or *Nr2f6*‐deficient (*Nr2f6^−/−^*) mice and anti‐Sirpα treated *Nr2f6*‐deficient (*Nr2f6^−/−^*) mice 72 h post‐*Salmonella* Typhimurium infection. F) Representative dot‐plots and quantification of frequencies of *S. aureus* Bioparticle phagocytosis into splenic macrophages of wild‐type (*Nr2f6^+/+^*) or *Nr2f6*‐deficient mice G) without or H) with pre‐treatment with CpG overnight, and I) relative induction of *S. aureus* Bioparticle phagocytosis. Representative data shown are from at least two independent experiments with *n* = 3‐6 (A‐E) and *n* = 2‐4 (F‐I) per group, treatment, and experiment, total *n* = 7/10/11 (*Nr2f6^+/+^* IgG1)/(*Nr2f6^‐/‐^* IgG1)/(*Nr2f6^‐/‐^* anti‐Sirpα) (A‐E), total *n* = 4/4/7/7 (*Nr2f6^+/+^* untreated)/(*Nr2f6^‐/‐^* untreated)/(*Nr2f6^+/+^* CpG treated)/(*Nr2f6^‐/‐^* CpG treated) (F‐I). Each dot represents the data from one individual mouse. Results are shown as median ± IQR with whiskers from min. to max. The Shapiro‐Wilk test evaluated the normality of data. Asterisks indicate statistically significant differences between genotypes calculated using either ordinary one‐way ANOVA/Kruskal‐Wallis Test for non‐parametric data or Student's *t*‐test/Mann‐Whitney *U* test for non‐parametric data. A *p*‐value < 0.05 was considered statistically significant. *0.05,**0.01, ***0.001, ****0.0001.

Anti‐Sirpα−treated *Nr2f6*‐deficient mice lost more weight than IgG‐treated controls, partially resembling the weight loss in wild‐type mice (**Figure** **8**A,B).

Splenic bacterial counts in anti‐Sirpα‐treated *Nr2f6*‐deficient mice increased but remained below wild‐type levels 72 h post‐infection (Figure 8C). Similarly, liver bacterial counts rose but did not reach wild‐type levels (Figure 8D).

We analyzed the percentage of Sirpα (CD172a)‐positive CD45^+^ blood cells to determine anti‐Sirpα antibody blocking efficiency. Consistent with prior data, *Nr2f6*‐deficient mice contained a significantly higher rate of CD172a‐positive cells, anti‐Sirpα‐treatment abolished Sirpα (CD172a)‐positive CD45^+^ blood cells (Figure 8E).

The P84‐antibody has shown moderate but significant inhibitory effects during tumor growth, particularly in combination therapies.^[^ ^60^, ^61^, ^62^ ^]^ We hypothesized that combined Sirpα/CD47 inhibition and CD36 activation via CpG oligodeoxynucleotide (CpG ODN), a TLR9 agonist, might enhance macrophage phagocytic capacity in *Nr2f6*‐deficient mice. CD36, a key scavenger receptor, facilitates pathogen and apoptotic cell phagocytosis and is upregulated by NR2F6 in the liver, which promotes hepatic steatosis.^[^ ^63^, ^64^, ^65^, ^66^, ^67^, ^68^ ^]^

CpG treatment modulates dendritic cell responses during *Salmonella* Typhimurium infection in vivo.^[^ ^69^, ^70^, ^71^ ^]^ We therefore examined splenic macrophage phagocytosis in *Nr2f6*‐deficient spleens in vitro using pHrodo™ red BioParticles conjugated with *Staphylococcus aureus* (*S. aureus*).

*Nr2f6*‐deficient macrophages showed reduced phagocytosis of *S. aureus* BioParticles (Figure 8F,G). Overnight CpG treatment restored the phagocytosis of *S. aureus* BioParticles by *Nr2f6*‐deficient macrophages to wild‐type levels, relative induction compared to wild‐type was significantly enhanced in *Nr2f6*‐deficient splenic cultures (Figure 8H,I).

In summary, anti‐Sirpα antibody treatment did not fully restore immune responses in vivo but influenced weight loss and bacterial loads in *Nr2f6*‐deficient mice. In vitro, both Sirpα blockade and CpG‐ treatment substantially enhanced phagocytosis *of Nr2f6*‐deficient macrophages.

## 3 Discussion

NRs are pivotal in macrophage development, inflammatory response, and host defense pathways.^[^ ^27^ ^]^ Our study is the first to describe the crucial involvement of NR2F6 in the regulation of tissue‐resident macrophages. While PPARγ is recognized for its essential role in alveolar macrophage and splenic RPM development and polarization, we observed no alterations in the expression of crucial RPM identity factors like Spi‐C, PPARγ, or genes associated with macrophage tissue retention such as VCAM‐1 or CD88 in *Nr2f6*‐deficient RPMs.^[^ ^5^, ^21^, ^33^ ^]^ In contrast to PPARγ‐deficient RPMs, which phagocytose stressed RBCs comparable to wild‐type at the single‐cell level, *Nr2f6*‐deficient macrophages mirror the functional reduction observed in *Spic*‐deficient mice, leading to an impaired sRBC phagocytosis.^[^ ^72^ ^]^

The observed increased Sirpα expression in *Nr2f6*‐deficient macrophages may suppress M_TR_ seeding by inhibiting migratory potential, as Sirpα negatively regulates β2 integrin‐mediated monocyte adhesion and transendothelial migration.^[^ ^55^, ^56^ ^]^ Nevertheless, other modalities might influence the seeding properties of *Nr2f6*‐deficient macrophages, such as the significantly enhanced expression of *Serpinb2*, which is known to inhibit the migration of peritoneal macrophages.^[^ ^73^ ^]^

While our studies underscore the role of NR2F6 in the regulation of Sirpα expression, which inhibits migration, phagocytosis and cytokine responses, we acknowledge the potential contribution of other regulatory mechanisms, including *Slc12a2* (NKCC1), *Cd300a*, and the signaling molecule *Inppl1* (SHIP‐2), which have been shown to contribute to phagocytic uptake.^[^ ^50^, ^53^, ^57^, ^74^, ^75^ ^]^ CD300a is especially critical in recognizing and clearing senescent, stressed, and apoptotic cells through efferocytosis under healthy conditions.^[^ ^76^, ^77^ ^]^ The impaired clearance of apoptotic cells in *Nr2f6*‐deficient mice could contribute to the late‐onset autoimmune syndrome observed in these mice.^[^ ^41b^ ^]^ Recent reports show that NR2F6 promotes hepatic steatosis in the liver through CD36 upregulation.^[^ ^65^ ^]^ CD36, a multifunctional class B scavenger receptor, is highly expressed in macrophages and plays a significant role in the recognition and phagocytosis of pathogens and apoptotic cells.^[^ ^66^, ^67^, ^68^ ^]^ Although *Cd36* expression in *Nr2f6*‐deficient splenic RPMs was not changed in our RNAseq dataset, we cannot exclude altered expression levels and subsequent functional alterations in other cells or tissues, such as the liver within the germline‐deficient mouse model.

Moreover, Sirpα on CD11c^+^ cells induces CD4^+^ Th17 cell differentiation and subsequent inflammation in the central nervous system during experimental autoimmune encephalomyelitis.^[^ ^78^ ^]^ We have previously shown that germline *Nr2f6*‐deficient mice are susceptible to Th17‐dependent autoimmune models.^[^ ^41b,d^ ^]^ Additionally, genetic ablation of *Nr2f6*, particularly in combination with the cancer immune checkpoint programmed cell death ligand 1 (PD‐L1) blockade, efficiently delays tumor progression by enhancing CD8 T cell responses.^[^ ^41c,f^ ^]^ Given recent evidence indicating that, during chronic infection, only Sirpα^+^ CD8^+^ T cells proliferate, transcribe IFNγ, and exhibit cytolytic activity, and because therapeutic blockade of PD‐L1 expands especially this cytotoxic subset of Sirpα^+^ CD8^+^ T cells,^[^ ^79^ ^]^ further investigations into the functional relationship of NR2F6 and Sirpα expression also in other immune cells will be of interest.

In human HEK cells, NR2F6 is involved in the transcription factor (TFs) network regulation of cytokine/chemokine gene promoters and activates cytokine expression.^[^ ^36^ ^]^ Luciferase assays revealed enhanced expression of *CCL15*, *CCL23*, *CCL24*, *CCL28*, *CXCL3*, *IFNA8*, *IFNA16*, *IL7*, *IL12B*, and *IL17A* in the human cell line HEK 293T co‐transfected with an NR2F6 expressing vector.^[^ ^36^ ^]^ Conversely, siRNA knockdown of NR2F6 in primary human macrophages, followed by LPS activation, reduced the expression of CCL15, CXCL3, and IL12B.^[^ ^36^ ^]^ *Salmonella* Typhimurium infection of our germline *Nr2f6*‐deficient animal model revealed significantly diminished plasma cytokine responses. Similarly, cytokine responses in *Nr2f6*‐deficient bone marrow‐derived macrophages were reduced, contrary to recent data indicating that NR2F6 silencing enhances the expression of inflammatory cytokines such as *Il1b*, *Ifng*, and *tumor necrosis factor (Tnf)a* within mouse BMDMs.^[^ ^37^ ^]^ In viral innate immunity, NR2F6 promotes HSV‐1 replication and gene expression via binding to the promoter of MAP3K5 and activating AP‐1/c‐Jun pathway, which is critical for DNA virus replication. On the other hand, NR2F6 is transcriptionally repressed by c‐Jun and forms a negative feedback loop. Meanwhile, cGAS/STING innate immunity signaling represses NR2F6 through STAT3.^[^ ^80^ ^]^

Our findings indicate that the combination of reduced splenic macrophage cell numbers in *Nr2f6*‐deficient mice, with decreased phagocytic potential due to enhanced Sirpα, contributes to impaired activation and pro‐inflammatory cytokine production in infected macrophages following *Salmonella* Typhimurium infection. As inside macrophages, *Salmonella* Typhimurium manipulates the host cell environment to promote survival and proliferation, it is protected from extracellular immune mechanisms. Subsequently, the reduced phagocytosis observed in the macrophages of *Nr2f6*‐deficient mice protects them from intracellular replication, bacterial spreading, and inflammatory processes that further enhance iron storage in the macrophages. Iron's decreased availability has been uncovered as a central mechanism hindering intracellular *Salmonella* Typhimurium multiplication while promoting anti‐bacterial immune effector mechanisms.^[^ ^20^, ^81^ ^]^

In addition, non‐classical monocyte‐derived CD9^+^ macrophages also exhibit significantly enhanced Sirpα surface receptor levels. This reduction in phagocytic potential via increased Sirpα on both splenic macrophages and CD9^+^ macrophages, contributes to the reduced susceptibility of *Nr2f6*‐deficient mice to *Salmonella* Typhimurium infection.

Along this line, SIRPα‐deficient (*Sirp*α*^−/−^*) mice are highly susceptible to *Salmonella* Typhimurium and succumb to systemic infection with attenuated *Salmonella*, exhibiting significantly elevated bacterial loads in both the spleen and the liver.^[^ ^82^ ^]^ During viral infections, Sirpα functions as an anti‐viral entry factor targeting viruses using endocytic pathways, whereas in *Mycobacterium tuberculosis* infection, SIRPα maintains macrophage homeostasis by interacting with PTK2B kinase.^[^ ^83^, ^84^ ^]^ How, on a molecular mechanism, macrophage‐specific Sirpα contributes to the protection from *Salmonella* Typhimurium infection needs further investigation.

The recently identified mechanism of CD47 upregulation as a protective host checkpoint response to pathogen recognition could further lead to enhanced protection from *Salmonella* Typhimurium infection in *Nr2f6*‐deficient mice.^[^ ^85^ ^]^ NRs are essential regulators during *Salmonella* Typhimurium infection in mice. Like *Nr2f6*‐deficient mice, PPARδ−deficiency efficiently inhibits *Salmonella* Typhimurium replication, which is mechanistically linked to macrophage metabolic state and intracellular glucose availability.^[^ ^34^ ^]^ In parallel, mice lacking PPARα expressed lower levels of inflammatory cytokines, with reduced bacterial dissemination due to modulation of immunometabolism and macrophage polarization.^[^ ^28^ ^]^ In addition, the estrogen‐related receptor γ (EERγ) controls *Salmonella* Typhimurium infection by modulating host iron homeostasis.^[^ ^35^ ^]^ Similar to infected *Nr2f6*‐deficient mice, treatment with an inverse agonist of ERRγ, GSK5182, decreased *Hepcidin* mRNA expression and *Salmonella* Typhimurium proliferation in liver and spleen, restored plasma iron levels and enhanced survival of the mice.^[^ ^35^ ^]^ Currently, we cannot rule out whether the loss of NR2F6 in hepatocytes contributes to the protection against *Salmonella* Typhimurium infection. If treatment with GSK5182 further reduces *Salmonella* Typhimurium infection and survival of infected *Nr2f6*‐deficient mice will be investigated in future studies.

In parallel to the functional role of pro‐inflammatory cytokines and especially IL‐6 in liver hepcidin expression, TFR1 limits hepcidin induction by a homeostatic iron regulator (Hfe), an atypical major histocompatibility class 1 type molecule.^[^ ^86^ ^]^ As TFR1 protein levels in the spleen and the liver are prominently enhanced in *Salmonella* Typhimurium‐infected *Nr2f6*‐deficient mice, we cannot rule out a functional relationship in hepcidin regulation at the current stage.

Notably, *Nr2f6*‐deficient mice share similarities with IL‐6–knockout mice, exhibiting an inverse correlation between plasma iron levels and bacterial load. Currently, we cannot rule out the possibility that in germline *Nr2f6*‐deficient mice, other cells, such as basophils, eosinophils or dendritic cells, contribute to the reduced pro‐inflammatory cytokine levels. A reduction in *Salmonella* Typhimurium CFU occurs in the spleen and liver of both *Nr2f6*‐deficient and IL‐6–knockout mice, accompanied by stable plasma iron levels during infection.^[^ ^35^ ^]^ Future investigations, including analyzing a macrophage‐specific conditional *Nr2f6*‐deficient mouse model, will be essential to clarify these aspects in greater detail.

## 4 Conclusion

Collectively, our dataset presents evidence for the regulatory role of NR2F6 in tissue‐resident macrophage populations during homeostasis and bacterial infection.

Upon *Salmonella* Typhimurium infection, loss of NR2F6 protected mice from weight loss and bacterial burden in the spleen and the liver. This protection is partly attributed to the reduced phagocytic potential, driven by increased Sirpα expression on *Nr2f6*‐deficient splenic RPMs and monocyte‐derived CD9^+^ macrophages. Subsequently, pro‐inflammatory plasma cytokines such as G‐CSF, IL‐12, and IL‐6 and liver *Hepcidin* levels were reduced, altering host iron homeostasis and exerting a potent antimicrobial effect on the *Salmonella* Typhimurium infection, resulting in enhanced host survival.

## 5 Experimental Section

### Mouse Strains

The mice were bred and housed in the “Zentrale Versuchstieranlage (ZVTA)” in Innsbruck under specific pathogen‐free (SPF) conditions. The animals were maintained at room temperature, and water and food were provided ad libitum. Both male and female mice were used between 8 and 13 weeks of age. Mice were age‐matched for individual experiments. The ARRIVE guidelines (http://www.nc3rs.org.uk/ARRIVEpdf) were followed. All animal experiments were performed in accordance with national and European guidelines. The ethics committee of the Medical University of Innsbruck and the Austrian Federal Ministry of Education, Science and Research approved and authorized all animal procedures (GZ: 2021‐0.406.865; GZ: 2023‐0.483.705; GZ: 2024‐0.904.918). The *Nr2f6*‐deficient mice were received via an internal MTA from Gottfried Baier (Medical University of Innsbruck).

### Organ Harvest

Adult mice were terminally anesthetized by intraperitoneal (i.p.) injection of 200 µL Ketasol‐ (100 mg mL^−1^, Livisto)/ Xylasol (20 mg mL^−1^, Livisto) solution. Following the excision of the spleen, the mice were perfused from the right atrium using 0.9 % ice‐cold NaCl. Perfusion was terminated when the organs appeared clear and, hence, free of contaminating blood. Afterward, lungs and livers were harvested and processed for analysis.

### Splenocyte Extraction

Adult spleens were extracted and stored in 5 mL FACS‐A (PBS + 3 % fetal calf serum (FCS, Biowest) + 0.5 % Penicillin/Streptomycin (P/S, PAN Biotech)) on ice until processing. The spleens were dissected in 2 mL collection tubes filled with 1 mL digest medium (0.5 mg mL^−1^ Collagenase D (Roche) and 0.1 mg ml^−1^ DNase I (Roche)) using small scissors. Splenic pieces were then transferred into an additional 4 mL of digest medium and incubated for 30 min at 37°C. After digestion, splenic pieces were squashed against a 100 µm cell strainer using the end of a syringe plug.

Splenocyte suspension was centrifuged at 300 rcf for 6 min at 4°C. The cell pellet was resuspended in 2 mL Erythrocyte Lysis Buffer (ELB; 155 µm NH_4_Cl, 9.4 µm NH_4_HCO_3_, 1 mm EDTA, pH 7.4) and incubated for 5 min at room temperature (RT) to lyse contaminating red blood cells. The reaction was stopped with 4 mL FACS‐A and the cell suspension was filtered through a 40 µm filter.

Subsequently, 10 µL of the cell suspension was mixed with 1 µL Acridine Orange/Propidium Iodide (Logos Biosystems) stain for counting with the LUNA‐FL Dual Fluorescence Cell Counter (Logos Biosystems).

The rest of the cells were again centrifuged at 300 rcf for 6 min at 4 °C and resuspended in 1 mL FACS‐A. 1‐2 × 10^6^ cells per spleen were transferred into a U‐bottom plate (Falcon) for antibody staining, followed by flow‐cytometric analysis with the BD FACSCanto II.

### Liver and Lung Extraction

The liver processing protocol was adapted,^[^ ^87^ ^]^ whereas the protocols for lung harvest were adapted.^[^ ^46^ ^]^ The lung and one liver lobe were harvested from each mouse and stored on ice in 5 mL cold FACS‐A until processing.

The organs were weighed, cut in half, and dissected into small pieces in 2 mL collection tubes filled with 1 mL digest medium (0.5 mg mL^−1^ Collagenase D (Roche) and 0.1mg ml^−1^ DNase I (Roche)). Lung pieces were transferred into an additional 4 mL, liver pieces into 9 mL of digest medium, and incubated for 30 min at 37°C. Afterward, organ pieces were mashed through 100 µm cell strainers using the end of a syringe plug. Cell suspensions were transferred into a 15 mL tube. In the case of the liver, the cells were centrifuged at 50 rcf for 3 min at 4 °C to eliminate organ debris and dead cells. The supernatant was recovered into a fresh 15 mL tube and centrifuged at 300 rcf for 6 min at 4 °C. The cell pellet was resuspended in 4 mL FACS‐A, filtered through a 40 µm filter, and centrifuged again (300 rcf, 6 min, 4 °C). Finally, a defined number of cells was resuspended in 100 µL FACS‐A and transferred into a U‐bottom plate for surface antibody staining, followed by flow‐cytometric analysis. In the case of the lung and spleen, flow cytometry was performed with the BD FACSCanto II. Liver cells were acquired with the Aurora (Cytek). Cell counts were calculated after analysis using the live cell counts, the sample collection duration, and the sample flow rate. The staining procedure was conducted as described below.

### Adoptive Cell Transfer of Congenic Bone Marrow to Neonates

CD45.1^+^ BM cells (8 × 10^6^) were injected intra‐peritoneally in 50 µl PBS once to 3‐day‐old CD45.2 wild‐type or *Nr2f6*‐deficient pups. Mice were sacrificed 8–12 weeks later, and the CD45.1 donor‐derived cells were identified.^[^ ^42^ ^]^

### Flow Cytometry

Single‐cell suspensions were pre‐incubated with anti‐CD16/32 (BioLegend, #101302) for 10 min at 4 °C to block FcγIII/II receptors before surface staining. The cells were then incubated with fluorochrome‐conjugated or biotinylated monoclonal antibodies for 30 min at 4 °C. After washing with PBS, the Fixable Viability Stain 780 (BD Biosciences, #565388) or BD Horizon Fixable Viability Stain 575V (BD Bioscience, #565694) were used for 15 min at 4°C to stain dead cells. Flow‐cytometric analysis was conducted with BD FACSCanto II (BD Biosciences), Aurora (Cytek) or CytoflexS (Beckman‐Coulter), followed by data analysis with the FlowJo software (BD Biosciences, New Jersey, v10.8.1). Antibodies used: CD9‐PeCy7, MZ3, BioLegend, #124815,1:200; CD11b‐APC‐Cy7, M1/70, BioLegend, #101225, 1:200; CD11b‐PECy5.5, M1/70, BioLegend, #101227, 1:400; CD11b‐ PerCP Cy5.5, M1/70, eBiosciences), # A14787, 1:200; CD11c‐Biotin, N418, BioLegend, #117304, 1:200; CD11c PeCy7, N418, eBiosciences, #25‐0114‐82, 1:200; CD11c‐PeCy5.5, N418, BioLegend, #171328, 1:200; CD31‐BV785 390, BioLegend, #102435, 1:200; CD36‐APC HM36, BioLegend, #102612, 1:200; CD45‐BV650, 30‐F11, BioLegend, #103151, 1:200; CD45‐BV500, 30‐F11, BD, #561487, 1:200; CD45‐FITC, 30‐F11, eBiosciences, #11‐0451‐85, 1:200, CD45 PeCy7, 30 F11, BD, #561868, 1:200; CD45‐APC, 30‐F11, eBiosciences, #17‐0451‐81, 1:200; CD64‐FITC X54 5/7.1, BioLegend, #139315, 1:200; CD64 PE S18017D, BioLegend, #161003, 1:400; CD86 PeCy7, GL1, BioLegend, #105014, 1:200; CD88‐PeCy7 20/70, BioLegend, #135809, 1:200; CD106‐PE 429, BioLegend, #105713, 1:200; CD169‐PE‐Cy7 3D6.112, BioLegend, # 142411, 1:200, CD172a‐FITC P84, BioLegend, #144006, 1:200; CD206‐APC, C068C2, BioLegend, #141707, 1:100; ESAM‐PE, 1G8/ESAM, BioLegend, #136203, 1:200; F4/80‐BV510, BM8, BioLegend, #123135, 1:200; F4/80‐BV421, BM8, BioLegend, #123137, 1:200; F4/80‐PeCy7, BM8, BioLegend, #123114, 1:200; F4/80‐PE, BM8, BioLegend, #123109, 1:200; Ki‐67 –PE, 16A8, BioLegend, #652404, 1:200; Ly6C‐APC HK1.4, eBiosciences, #17‐5932, 1:200; Ly6C‐APC‐Cy7, HK1.4, BioLegend, #128025, 1:200; Ly6C‐FITC, HK1.4, BioLegend, # 128005, 1:200; Ly6C‐BV510, HK1.4, BioLegend, #128033, 1:200; Ly6G‐PE, 1A8, BioLegend, #127608, 1:200; Ly6G‐Pe‐Cy7, 1A8, BioLegend, #127618, 1:200; MHC‐II‐BV421 M5/114.15.2, BioLegend, #107632, 1:200; MHC II FITC, M5/114.15.2, eBiosciences, #11 5321‐81, 1:200; Siglec F‐BV421; E50‐2440, BD, #562681, 1:200; Tim‐4‐PeCy7, RMT4‐54, BioLegend, #130009, 1:200; Ter‐119 PE, Ter 119, BioLegend, #116207, 1:200; B220‐BiotinRA3‐6B2 Biolegend #103203, 1:400; CD3‐Biotin, 145‐2C11, BioLegend, #100304, 1:200; CD19‐Biotin 6D5, BioLegend, #115503, 1:200; NK1.1‐Biotin PK136, BioLegend, #108704, 1:200; Ly6G‐Biotin, 1A8, BioLegend, #127604, 1:200; Siglec‐F Biotin, S17007L BioLegend,, # 155512, Ter 119 ‐Biotin, Ter 119, BioLegend, #116204, 1:200.

### Immunohistochemistry

Mice were euthanized as described above. Spleen and liver were sampled, embedded into Tissue‐Tek OCT (Sakura), and frozen at ‐80 °C. Seven micrometers thick sections were cut with the Cryostat (Leica Biosystems), transferred onto Polysine slides (25 × 75 × 0.1 mm, ThermoFisher), and stored at ‐20 °C after drying at RT. For immunofluorescence staining, the slides were thawed at RT for 60 min. Next, the tissue sections were encircled with a PAP pen (Merck) to create a hydrophobic barrier between the samples. After the wax had dried, the tissues were fixed with acetone (Merck) for 10 min at RT. The slides were washed three times for 5 min with PBS, followed by blocking for 30 min with PBS + 1 % BSA (Sigma‐Aldrich) to minimize unspecific antibody binding. The blocking solution was washed away with PBS, and 30–50 µL antibody dilution (PBS + 1 % BSA), depending on the size of the tissue slice, was added and incubated for 30 min at RT in a humidified chamber. Antibodies used: F4/80‐PE (BM8, BioLegend, #123109) and CD11b‐FITC (M1/70, BioLegend, #101206) diluted 1:50. Afterward, the slides were washed three times for 5 min with PBS, rinsed thoroughly with tap water, and the tissue was mounted. A drop of Vectashield Vibrance Antifade mounting medium (Vector labs) was added to the tissue and covered with a coverslip. The mounting medium contained a DAPI dye to counterstain the nuclei. Mounted slides were stored at 4 °C for drying and microscopy the next day.

Microscopy images were captured with an Olympus IX70 inverted fluorescence microscope and a 10x and 40x water immersion objective. Scale bars were added to the images using ImageJ/Fiji software (NIH, Maryland, v1.53t). Confocal imaging was performed with an UltraVIEW VoX spinning disk confocal system connected to a Zeiss AxioObserver Z1 microscope. (Figure 3J, 40x water immersion objective).

### Bone Marrow Extraction

The protocol for BMDM differentiation was adapted.^[^ ^88^ ^]^ The tibia and femur of both legs were excised. The bones were stored in 5 mL FACS‐A on ice until processing. The bones were disinfected in 70 % ethanol in a cell culture hood and washed in sterile FACS‐A. Each epiphysis cut off, and the bones were transferred into a 0.5 mL reaction tube with a hole in the bottom, which was placed into a 1.5 mL reaction tube. The tubes were centrifuged at 3000 rcf for 20 s at 4°C, after which the bone marrow cells accumulated at the bottom of the 1.5 mL tube. Erythrocytes were lysed by incubation with 2 mL ELB for 5 min at room temperature. Afterward, the cells were washed with sterile FACS‐A, filtered through a 40 µm filter, centrifuged again, and then counted using the LUNA‐FL Dual Fluorescence Cell Counter. The cell suspension was either frozen or directly seeded for BMDM differentiation.

### Differentiation of Bone Marrow‐Derived Macrophages

Fresh or frozen bone marrow cells were used to generate BMDM. Cells from one mouse were either seeded into a 10 cm cell culture dish for different assays or nine wells of a 12‐well plate for stimulation. In the case of a 10 cm dish, 4 × 10^6^ bone marrow cells were resuspended in 10 mL BMDM differentiation media, consisting of RPMI (M&B Stricker) supplemented with 10 % FCS, 1 % L‐glutamine (PAN Biotech), 1 % P/S (PAN Biotech), 0.05 mm β‐mercaptoethanol (Sigma‐Aldrich) and 40 ng mL^−1^ M‐CSF (Peprotech, #315‐02). For seeding into a 12‐well plate, 5 × 10^5^ cells per well were resuspended in 1 mL BMDM differentiation media. In both cases, the cells were incubated for three days at 37 °C, 5 % CO_2_ before half of the medium per well or plate was replaced with fresh BMDM differentiation medium. The cells were incubated for three more days at 37 °C, 5 % CO_2_. On day six of culture, all the media were replaced with fresh BMDM differentiation meda. The cells were replated for some assays on day six of culture. For stimulation, the cells were cultured until day seven and afterward stimulated (described below).

### BMDM Stimulation and Polarization

After seven days of culture in 12‐well plates, the BMDM medium was removed, and the cells were serum‐starved for 2 h with 0.5 mL of X‐Vivo media (Lonza). Afterward, the BMDMs were left untreated (medium control) or were treated with LPS (100 ng mL^−1^, Sigma‐Aldrich, #L4391) plus IFNγ (50 ng mL^−1^, Peprotech, #315‐05) or IL‐4 (20 ng µL^−1^, Peprotech, #214‐14) for 4 or 24 h. After stimulation, three of the wells seeded per mouse were used for cell lysis and subsequent mRNA isolation, as described below. After 24 h, the remaining wells were used to obtain the BMDMs for flow‐cytometric analysis. First, 200 µL of the supernatant from each well was harvested and immediately frozen at ‐20 °C for later Bioplex analysis. The rest of the media was discarded, and the attached cells were washed with 1 mL pre‐warmed HBSS. Next, 300 µL of TrypLE (Gibco) was added to each well and incubated at 37°C for 10 min. The reaction was stopped by adding 700 µL of FACS‐A, and macrophages were detached by pipetting the solution across the dish. The cells were collected in 15 mL tubes and centrifuged at 200 rcf, 7 min at 4°C. Afterward, all the cells were resuspended in 100 µL FACS‐A and transferred into a 96‐well U‐bottom plate for surface antibody staining, followed by flow cytometry. The staining procedure was conducted as described above.

### RNA Isolation and Quantitative Real‐Time PCR (qRT‐PCR)

Untreated BMDMs or BMDMs treated with LPS + IFNγ or IL‐4 for 4 or 24 h were washed with PBS once and then directly lysed with RLT (Qiagen). Lysed cells were stored at ‐80°C until RNA isolation. The cell lysates were thawed for 15 min at 37°C, and total RNA was extracted using the RNeasy Mini Kit (Qiagen) according to the manufacturer's instructions. Next, the RNA quality and concentration were determined by spectrophotometry (DeNovix DS‐11), and RNA was reverse‐transcribed into cDNA using the Omniscript RT Kit (Qiagen) and oligo (dT) primers (Promega). Reverse transcription was performed according to the supplier's instructions. cDNA was stored at ‐20°C until analysis. Gene expression analysis for *iNos*, *Il6*, or *Arg1*, was performed using Blue S'Green qPCR Kit (Biozym) compared with *Actb* (β‐actin). The primers were diluted to a concentration of 10 µm prior to use. SYBR green primer sequences. Amplifications were set up in duplicates in a 96‐well PCR plate (Biozym), and non‐template controls were included to detect possible contaminations. Primer (mouse) Sequences: *Actb*: Fw 5′ GACGGCCAGGTCATCACTATTG 3′, Rv 5′ AGGAAGGCTGGAAAAGAGCC 3′ (130bp); *iNos*: Fw 5′ CACCAAGCTGAACTTGAGCG 3′, Rv 5′ CGTGGCTTTGGGCTCCTC 3′ (124bp); *Arg1*: Fw 5′ CTCCAAGCCAAAGTCCTTAGAG 3′, Rv 5′ GGAGCTGTCATTAGGGACATCA 3′ (180bp); *Il6*: Fw 5′ TGTGCAATGGCAATTCTGAT 3′, Rv 5′ GGTACTCCAGAAGACCAGAGGA 3′ (155bp) Following primer were used for gene expression analysis in spleen and liver: *Hamp*: Fw 5′ GGCAGACATTGCGATACCAAT 3′, Rv 5′ GCAACAGATACCACACTGGGA 3′, probe CCAACTTCCCCATCTGCATCTTCTGC; *Fth1* Fw 5′ GCGAGGTGGCCGAATCT 3′, Rv 5′ CAGCCCGCTCTCCCAGT 3′, probe CCAACTTCCCCATCTGCATCTTCTGC; *Lcn2* Fw 5′ GCCTCAAGGACGACAACATCA 3′, Rv 5′ CACCACCCATTCAGTTGTCAAT 3′, probe TTCTCTGTCCCCACCGACCAATGC; *Tfr1* Fw 5′ CGCTTTGGGTGCTGGTG 3′, Rv 5′ GGGCAAGTTTCAACAGAAGACC 3′; probe CCCACACTGGACTTCGCCGCA; *Hprt* Fw 5′ GACCGGTCCCGTCATGC 3′, Rv 5′ TCATAACCTGGTTCATCATCGC 3′, probe TTCTCTGTCCCCACCGACCAATGC.

Gene‐specific amplification was performed with the 7500 Fast Real‐Time PCR System (Applied Biosystems). For analysis, delta‐delta cycle threshold (ddCT) values were calculated in relation to the housekeeping gene (*Actb*, *Gapdh, or Hprt*), and expression fold changes of the respective genes were calculated relative to, e.g., uninfected wild‐type or unstimulated BMDMs.

### Cytokine Measurements

CXCL1, TGF‐β1 (Free Active), IL‐18, IL‐23, MDC (A8), IL‐10, IL‐12p70, IL‐6, TNFα, G‐CSF, TARC, IL‐12p40, IL‐1β cytokine levels from mouse plasma or cell culture supernatants were analyzed with LEGENDplex Mouse Macrophage/Microglia Panel (13‐plex) Kit (BioLegend, #740846) according to the instruction manual.

### Bacteria Strains

*Salmonella enterica* serovar Typhimurium (ATCC 14028) and *Salmonella enterica* serovar Typhimurium strain SL1344 expressing red fluorescent protein (RFP) (*Salmonella* Typhimurium ^(red)^) were used according to established protocols.^[^ ^89^, ^90^ ^]^

### In Vitro Salmonella Typhimurium Infection of BMDMs and Splenocytes

A pre‐culture of *Salmonella* Typhimurium (ATCC 14028 or SL1344 expressing RFP) in Lysogeny Broth (LB, ROTH, Karlsruhe, Germany) medium was shaken at 37 °C overnight. The following day, 50 µL of the bacterial suspension was transferred into 10 mL of LB medium and shaken for ≈2 h at 37 °C to reach an optical density of 0.5 at 600 nm (OD600). This value was set to ensure the bacteria were in the logarithmic growth phase. Viable *Salmonella* Typhimurium was counted using the Casy counting system (OMNI Life Science, Bremen, Germany). Cells were infected for 30, 60, 90, or 180 min with a multiplicity of infection (MOI) of 10. Afterward, *Salmonella* Typhimurium, which was not phagocytosed, was washed away with PBS + 25 mg mL^−1^ gentamicin (Gibco, Darmstadt, Germany), and 1 mL of DMEM media (10% FBS + 1% L‐glutamine + 25 mg mL^−1^ gentamicin) was added to each well.^[^ ^89^, ^90^ ^]^ Intracellular *Salmonella* Typhimurium was determined by flow cytometry or microscopic analysis as described above.

### Bacteria‐Induced Infection Model

In vivo infection with *Salmonella* Typhimurium (ATCC 14028) was carried out as described.^[^ ^81^ ^]^ Specifically, 1,000 live *Salmonella* Typhimurium in 200 µL PBS were i.p. injected into 8‐12‐week‐old mice. Blood samples were taken 1 day and 3 days post‐infection for cytokine measurement, and plasma was collected. In each in vivo assay, weight and surface body temperature were measured in at least 12 h intervals. The loss of reflexes (righting and grabbing reflex) and/or a body temperature drop of the animal of more than 5 °C or weight loss below 20% compared with the pre‐infection baseline was determined as an endpoint for infection experiments.^[^ ^89^ ^]^

### Anti‐Sirpα Treatment

In vitro before *Salmonella* Typhimurium infection, total splenocytes were incubated for 30 min. in RPMI medium with or without 5 µg mL^−1^ InVivoMAb anti‐mouse CD172a (P84, BioXcell, #BE0322). Following incubation, the medium was removed, and the cells were washed once with PBS before being immediately infected. In vivo, mice were i.p. injected with 100 µg InVivoMAb anti‐mouse CD172a (P84, BioXcell, #BE0322) in PBS or 100 µg anti‐IgG antibody (HTK888, BioLegend, #400959) in 150 µl PBS one day prior to *Salmonella* Typhimurium infection, as well as on days 1 and 2 post‐infection.

### Colony Forming Units

The bacterial load in the blood, liver, and spleen was determined by plating serial dilutions of homogenized infected organ pieces onto LB agar plates under sterile conditions. For in vitro experiments, BMDMs were lysed with 1 mL of 0.5% sodium deoxycholic acid (Sigma‐Aldrich). Serial dilutions of lysate were plated on LB agar plates. The colonies were counted manually after a 12 h incubation of the plates at 37 °C. For lysates of organs, the number of bacteria per gram of tissue was calculated; for cell culture and blood, the CFU per mL was determined.^[^ ^89^, ^90^ ^]^

### Plasma Iron

Plasma iron concentrations were measured with a colorimetric iron quantification kit (QuantiChrom Iron Assay Kit, BioAssay Systems) following the manufacturer's instructions.^[^ ^91^ ^]^

### Western Blot Analysis

Protein extraction and Western blotting were performed as described^[^ ^22^ ^]^ using a rabbit anti‐FPN1 antibody (1:2000; self‐made, MW: 66 kDa), a rabbit anti‐FRT (Ferritin) antibody (1:500; Sigma Aldrich, MW: 20 kDa), a mouse anti‐TFR1 (1:1000; Sigma Aldrich, MW: 100 kDa), a rabbit anti‐ACTB antibody (1:500; Sigma Aldrich, MW: 45 kDa).

### Intracellular Iron

Following a gentamycin protection assay, splenocytes were incubated with 1 µmol L^−1^ FerroOrange (DOJINDO, F374) in PBS at 37°C for 30 min. Fluorescence was subsequently analyzed using a TECAN Spark microplate reader, as previously described.^[^ ^92^ ^]^ Fluorescence intensity was normalized to unstained and uninfected controls.

### Cell Sorting, RNA Preparation, and RNA‐Seq

Splenic single‐cell suspension of wild‐type or *Nr2f6*‐deficient mice was pre‐incubated with 1 µg ml^−1^ of αCD16/32 Fc‐Block (BioLegend, 101302) for 15 min and stained with life/death marker (fixable viability stain 780, BD Biosciences). Subsequently, cells were stained with CD64‐FITC (X54‐5/7.1, BioLegend), CD106‐PE (429 (MVCAM.A), BioLegend), CD11b‐PerCP Cy5.5 (M1/70, eBiosciences), CD169‐PE‐Cy7 (3D6.112, BioLegend), CD45‐APC (30‐F11, eBiosciences), CD3‐Biotin (17A2, BioLegend), NK1.1‐Biotin (PK136, BioLegend), CD19‐Biotin (6D5, BioLegend), B220‐Biotin (RA3‐6B2, BioLegend), Ly6‐G‐Biotin (1A8, BioLegend), Ter‐119‐Biotin (Ter‐119, BioLegend), and Siglec‐F (S17007L, anti BioLegend) antibody cocktail for 30 min. followed by Streptavidin Alexa Flour 700 conjugate (ThermoFisher Scientific) staining for 30 min. RPMs were sorted as live/CD45^+^/Lin^‐^CD11b^mid^/F4/80^+^/CD64^‐^/CD169^‐^/CD106^+^ on a FACS Aria III (BD Biosciences). Non‐singlet events were excluded from analyses based on characteristics of FSC‐H/FSC‐W and SSC‐H/SSC‐W. RNA from sorted cells was extracted using the RNeasy mini Kit as per manufacturer instructions. DNA was removed using DNA‐removal columns (Quiagen). RNA quantity and integrity were determined by Agilent 5400, and only samples with > 100 ng RNA and RNA integrity number > 4 were used.

mRNA was isolated from total RNA using poly‐T oligo‐attached magnetic beads. The isolated mRNA was subjected to fragmentation, followed by the synthesis of first‐strand cDNA employing random hexamer primers. Subsequently, second‐strand cDNA synthesis was performed with dTTP to create a non‐directional library. The prepared library underwent a series of steps, including end repair, poly‐A enrichment, adapter ligation, size selection, amplification, and purification. The library quality and quantity were assessed using Quibit and real‐time PCR for quantification and a bioanalyzer for size distribution analysis. Quantified libraries were pooled and subjected to next‐generation Illumina sequencing generating paired‐end reads.

Clean reads were obtained by filtering raw reads to remove those containing adapters, reads with more than 10% undetermined bases, and low‐quality reads (quality value of over 50% bases of the read is ≤5) using the fastp software. Paired‐end clean reads were aligned to the mouse genome (mm10) using HISAT2 (V.2.0.5).^[^ ^93^ ^]^ Gene counts were derived based on the number of reads mapped to each gene using featureCounts (v1.5.0‐p3). Differentially expression (DE) analysis was performed using the DESeq2 R package (1.20.0). The resulting p‐values were adjusted using Benjamini and Hochberg's approach to control the false discovery rate. Genes with an adjusted P‐value ≤0.05 were considered as differentially expressed (DEG).

### Red Blood Cell Phagocytosis Assay

Spleens of wild‐type and *Nr2f6*‐deficient mice were harvested and processed as described above. Blood from one wild‐type mouse was collected in 10 µL heparin to avoid clotting and centrifuged at 1000 rcf for 10 min at 4°C. The serum was removed, and RBCs were washed with 1 mL HBSS (Gibco), centrifuged again, and resuspended in 500 µL HBSS. RBCs were placed at 48°C for 30 min with shaking to generate stressed RBCs. Afterward, the sRBCs were diluted 1:1000 in HBSS for counting using a Neubauer Chamber. Splenocytes were transferred into a 96‐well plate at a density of 1 x 10^6^ cells per well. 1 x 10^7^ sRBCs were added per well to reach a ratio of 10:1. Control cells were left without sRBCs. The cells were incubated for 1.5 h at 37°C and 5% CO_2_. Following incubation, the cells were immediately put on ice to stop phagocytosis. Before surface staining, excess sRBCs were lysed for 5 min by adding 50 µL ELB at 4 °C. The cells were washed, stained and measured by flow cytometric analysis.

### Bioparticle Phagocytosis Assay

Single‐cell suspensions of splenocytes were prepared and seeded as described for the RBC phagocytosis assay. For phagocytosis, pHrodo Red Zymosan, or *S*. *aureus*, BioParticles were used in a live cell imaging solution. BioParticles were added at 1:10 to 100 µL of single‐cell suspension for an end concentration of 0.1 mg mL^−1^. The cells were incubated for 1.5 h at 37 °C and 5% CO_2_. Following incubation, the cells were immediately placed on ice to stop phagocytosis. The cells were washed and the staining procedure was conducted as described above, followed by flow cytometric analysis.

### Software and Algorithms

FlowJo 10; RRID:SCR_008520 (TreeStar); Fiji (ImageJ) http://fiji.sc; RRID:SCR_002285 N/A; Prism 10 (https://www.graphpad.com/; RRID:SCR_002798) Graphpad; R (https://www.r‐project.org/).

### Declaration of Generative AI and AI‐Assisted Technologies in the Writing Process

During the preparation of this work, the author(s) used Grammarly and ChatGPT to improve language and readability. After using this tool/service, the author(s) reviewed and edited the content as needed and take(s) full responsibility for the publication's content.

### Statistical Analysis

Statistical analysis was performed using Prism 10.2.3. Unless otherwise indicated, experiments were repeated at least two times using a minimum of 3 mice per group ‐ the Shapiro–Wilk normality test evaluated the data's normality. When normally distributed, statistical analysis was performed with an unpaired Student's *t*‐test or the one‐way ANOVA for samples with equal variance (*F* test) or an unpaired *t*‐test with Welch's correction for datasets with different variances. A Mann–Whitney U or Kruskal‐Wallis test was used if data were not normally distributed. Results are shown as median ± IQR with whiskers from min. to max, or mean ± SEM. Outliers were excluded using ROUT (Q = 1%) in GraphPad Prism. A *p*‐value < 0.05 was considered statistically significant. *p < 0.05; **p < 0.01, ***p < 0.001. Randomization, blinding, or sample size estimation tests were not applied to the animal studies.

## Conflict of Interest

The authors declare no conflict of interest.

## Author Contributions

This study was designed by N.H.‐K. J.W., C.‐P.O., J.B., N.B., and N.H.‐K. performed most experiments. C.‐P.O., N.B., J.W., and N.H.‐K. performed in vitro and in vivo bacterial infection models. J.W. performed the cell sort and RNA‐Seq analysis. A.K. and M.H. set up and analyzed immunohistological experiments and performed microscopical analyses. C.‐P.O., N.B., M.B., J.B., J.W., and N.H.‐K. performed phagocytosis experiments. J.B., J.W., C.P.‐O., N.B., M.B., A.K., and N.H.‐K. analyzed and interpreted data and designed figures. G.W. gave intellectual input and helped with concepts. N.‐H.K. wrote the first draft, and all authors read, edited, and approved the final version of the manuscript.

## Supporting information



Supporting Information

## Data Availability

This study did not generate new datasets or code. RNAseq primary data deposition is found under GEO accession GSE250198: https://www.ncbi.nlm.nih.gov/geo/query/acc.cgi?acc=GSE250198. All data are available in the main text or the supplementary materials. The *Nr2f6*‐deficient mice were received via an internal MTA from Gottfried Baier (Medical University of Innsbruck).
