# Supplementary material for: Loss of NR2F6 Protects from Salmonella Typhimurium Infection
Source: Adv Sci (Weinh). 2025 Jul 2;12(37):e04280. doi: 10.1002/advs.202404280 (PMC12499465; doi:10.1002/advs.202404280)
Supplement: Supplementary file 1 — Supporting Information [file ADVS-12-e04280-s001.pdf]

ADVANCED SCIENCE
Open Access

Supporting Information

for *Adv. Sci.*, DOI 10.1002/advs.202404280

Loss of NR2F6 Protects from *Salmonella* Typhimurium Infection

*Johannes Woelk, Christa Pfeifhofer-Obermair, Julia Benz, Natascha Brigo, Milena Bamberger, Alexeja Kleiter, Martin Hermann, Guenter Weiss and Natascha Hermann-Kleiter**

Loss of NR2F6 Protects from *Salmonella* Typhimurium Infection

*Johannes Woelk, Christa Pfeifhofer-Obermair, Julia Benz, Natascha Brigo, Milena Bamberger, Alexeja Kleiter, Martin Hermann, Guenter Weiss, Natascha Hermann-Kleiter**

J. Woelk, M, J. Benz,. Bamberger, A. Kleiter, N. Hermann-Kleiter

Institute of Cell Genetics, Department for Genetics, Medical University of Innsbruck, Innsbruck 6020, Austria.

E-mail: (natascha.kleiter@i-med.ac.at (N-HK))

J. Benz,

Currently, Institute of Microbiology – Clinical Microbiology, Immunology and Hygiene, University Clinic Erlangen, Friedrich-Alexander-University Erlangen-Nuremberg, Erlangen 91054, Germany

C. Pfeifhofer-Obermair, N. Brigo, G. Weiss,

Department of Internal Medicine II (Infectious Diseases, Immunology, Rheumatology, Pneumology), Medical University of Innsbruck 6020, Austria.

A. Kleiter

Currently, Laboratory for Langerhans Cell Research, Department of Dermatology, Venereology & Allergology and 3D Bioprinting Laboratory, Department of Pediatrics I, Medical University Innsbruck 6020, Austria

M. Hermann

Department of Anaesthesiology and Critical Care Medicine, Medical University of Innsbruck 6020, Austria.

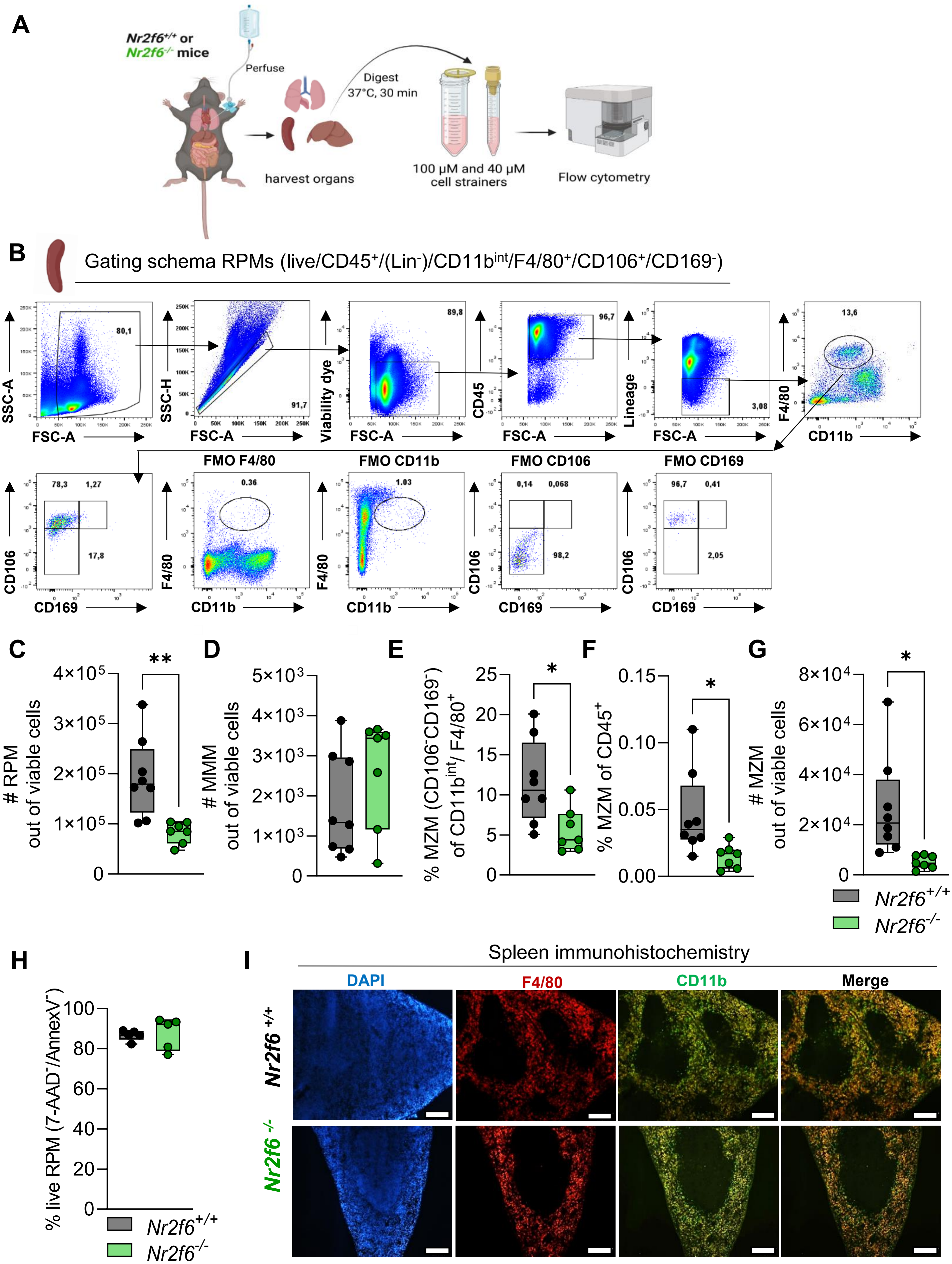

A
Nr2f6+/+ or Nr2f6-/- mice
Perfuse
harvest organs
Digest 37°C, 30 min
100 µM and 40 µM cell strainers
Flow cytometry
B
Gating schema RPMs (live/CD45+/(Lin-)/CD11bint/F4/80+/CD106+/CD169-)
SSC-A
FSC-A
SSC-H
Viability dye
CD45
Lineage
F4/80
CD11b
CD106
CD169
FMO F4/80
FMO CD11b
FMO CD106
FMO CD169
C
RPM out of viable cells
D
MMM out of viable cells
E
% MZM (CD106-CD169-) of CD11bint/ F4/80+
F
% MZM of CD45+
G
MZM out of viable cells
Nr2f6+/+
Nr2f6-/-
H
% live RPM (7-AAD-/AnnexV-)
I
Spleen immunohistochemistry
DAPI
F4/80
CD11b
Merge

Supplemental Figure 1: Characterization of tissue resident macrophage populations in *Nr2f6*-deficient spleens. **(A)** Scheme depicting the experimental procedure of *Nr2f6*$^{+/+}$ or *Nr2f6*$^{-/-}$ organ harvest following anesthesia and perfusion with 0.9 % NaCl. Spleens, livers, and lungs were harvested, weighed, digested, and processed into single-cell suspensions for flow-cytometric analysis (Created with BioRender.com). **(B)** Dot plots of one representative spleen show the gating strategy to identify splenic macrophage subsets. Following double exclusion cells were gated on live, CD45^{+}, Lin^{-}, splenic macrophages (CD11b$^{\mathbf{int}}$/ F4/80$^{\mathbf{+}}$) and subsequent gating on red pulp (RPM: CD106^{+}CD169^{-}), marginal metallophilic (MMM: CD106^{+}CD169^{+}) and marginal zone macrophages (MZM: CD106^{-}CD169^{-}), FMO controls for F4/80 , CD11b, CD106 and CD169 are included. **(C)** Quantification of RPM and **(D)** MMM total cell numbers in the spleen of wildtype (*Nr2f6*$^{+/+}$) or *Nr2f6*-deficient (*Nr2f6*$^{-/-}$) mice. **(E)** Quantification of MZM frequencies within CD11b$^{\mathbf{int}}$/ F4/80$^{\mathbf{+}}$ macrophages, **(F)** frequencies of CD45 and **(G)** total cell numbers in the spleen of wildtype (*Nr2f6*$^{+/+}$) or *Nr2f6*-deficient (*Nr2f6*$^{-/-}$) mice.
(H) Frequencies of splenic adult wild-type (*Nr2f6*$^{+/+}$) or *Nr2f6*-deficient (*Nr2f6*$^{-/-}$) living (7-AAD^{-}/Annexin V^{-}) red pulp macrophages. **(I)** Two-color immunohistofluorescence staining of adult wild-type (*Nr2f6*$^{+/+}$) or *Nr2f6*-deficient (*Nr2f6*$^{-/-}$) spleens, fixed and incubated with anti-F4/80 and anti-CD11b antibodies. Merged images show co-localized red and green fluorescence (RPMs, orange areas). Nuclei were counterstained with DAPI (blue). A 10-fold magnification overview is shown, scale bars, 200 µm. Representative data shown are from at least two independent experiments with *n* = 2-4 per group and experiment, total *n* = 8/8 (*Nr2f6*$^{+/+}$)/ (*Nr2f6),* and *n* = 4/4 (for histological sections) (*Nr2f6*$^{+/+}$)/(*Nr2f6*$^{-/-}$). Each dot represents the data from one individual mouse. Results are shown as median ± IQR with whiskers from min. to max. The Shapiro-Wilk test evaluated the normality of data. Asterisks indicate statistically significant differences between genotypes calculated using the Student's *t*-test or Mann-Whitney *U* test for non-parametric data.

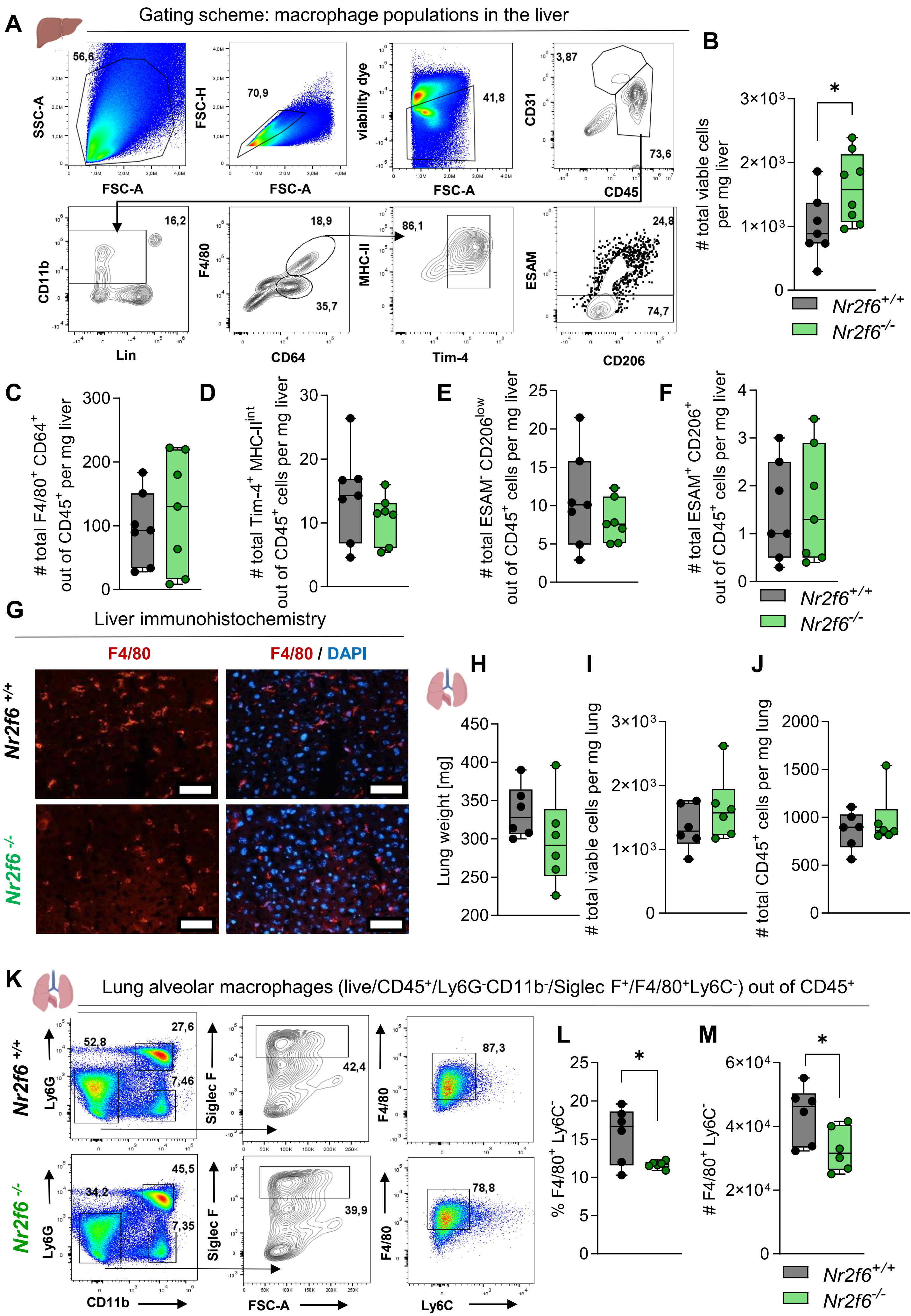

A
Gating scheme: macrophage populations in the liver
B
C
D
E
F
G
Liver immunohistochemistry
F4/80
F4/80 / DAPI
Nr2f6 +/+
Nr2f6 -/-
H
I
J
Lung weight [mg]
total viable cells per mg lung
total CD45+ cells per mg lung
K
Lung alveolar macrophages (live/CD45+/Ly6G-CD11b-/Siglec F+/F4/80+Ly6C-) out of CD45+
L
M
% F4/80+ Ly6C-
# F4/80+ Ly6C-
# total viable cells per mg liver
# total F4/80+ CD64+ out of CD45+ per mg liver
# total Tim-4+ MHC-IIint out of CD45+ cells per mg liver
# total ESAM- CD206low out of CD45+ cells per mg liver
# total ESAM+ CD206+ out of CD45+ cells per mg liver
Nr2f6+/+
Nr2f6-/-
SSC-A
FSC-A
FSC-H
viability dye
CD31
CD45
CD11b
Lin
F4/80
CD64
MHC-II
Tim-4
ESAM
CD206
Ly6G
Siglec F
Ly6C

Supplemental Figure 2: Loss of *Nr2f6* leads to alterations of liver and lung tissue-resident macrophages. **(A)** Dot- and contour plots of one representative liver showing the gating strategy to identify liver-resident macrophages and Kupffer cell subsets. Macrophages were identified as $CD45^{+}Lin^{-}CD11b^{+}F4/80^{+}CD64^{+}$. Kupffer cells (KC) were defined as $CD45^{+}Lin^{-}CD11b^{+}F4/80^{+}CD64^{+}$Tim-4 MHC-$II^{int}$, KC1 additionally express $ESAM^{-}$ $CD206^{lo}$ and KC2 $ESAM^{+}$ $CD206^{+}$. **(B)** Quantification of total viable cells, **(C)** liver macrophages, **(D)** Kupffer cells, **(E)** KC1, and **(F)** KC2 cells per mg liver of wild-type ($Nr2f6^{+/+}$) or *Nr2f6*-deficient ($Nr2f6^{-/-}$) mice. **(G)** Two-color immunohistofluorescence staining of adult wild-type ($Nr2f6^{+/+}$) or *Nr2f6*-deficient ($Nr2f6^{-/-}$) livers, fixed and incubated with anti-F4/80 antibody. Nuclei were counterstained with DAPI (blue). A 40-fold magnification overview is shown, scale bars, 50 µm. **(H)** Quantification of lung weight, **(I)** total viable cells, **(J)** and total $CD45^{+}$ cells per mg lung of wild-type ($Nr2f6^{+/+}$) or *Nr2f6*-deficient ($Nr2f6^{-/-}$) mice. **(K)** Representative dot-plots and **(L)** quantification of frequencies of alveolar macrophages **(M)** and total cell numbers per mg lung of wild-type ($Nr2f6^{+/+}$) or *Nr2f6*-deficient ($Nr2f6^{-/-}$) mice. Representative data shown are from three independent experiments with n = 2 per group and experiment, total n = 6/6 ($Nr2f6^{+/+}$)/(*Nr2f6).* Each dot represents the data from one individual mouse. Results are shown as median ± IQR with whiskers from min. to max. The Shapiro-Wilk test evaluated the normality of data. Asterisks indicate statistically significant differences between genotypes calculated using the Student's t-test or Mann-Whitney U test for non-parametric data. A p-value < 0.05 was considered statistically significant. *$p < 0.05$.

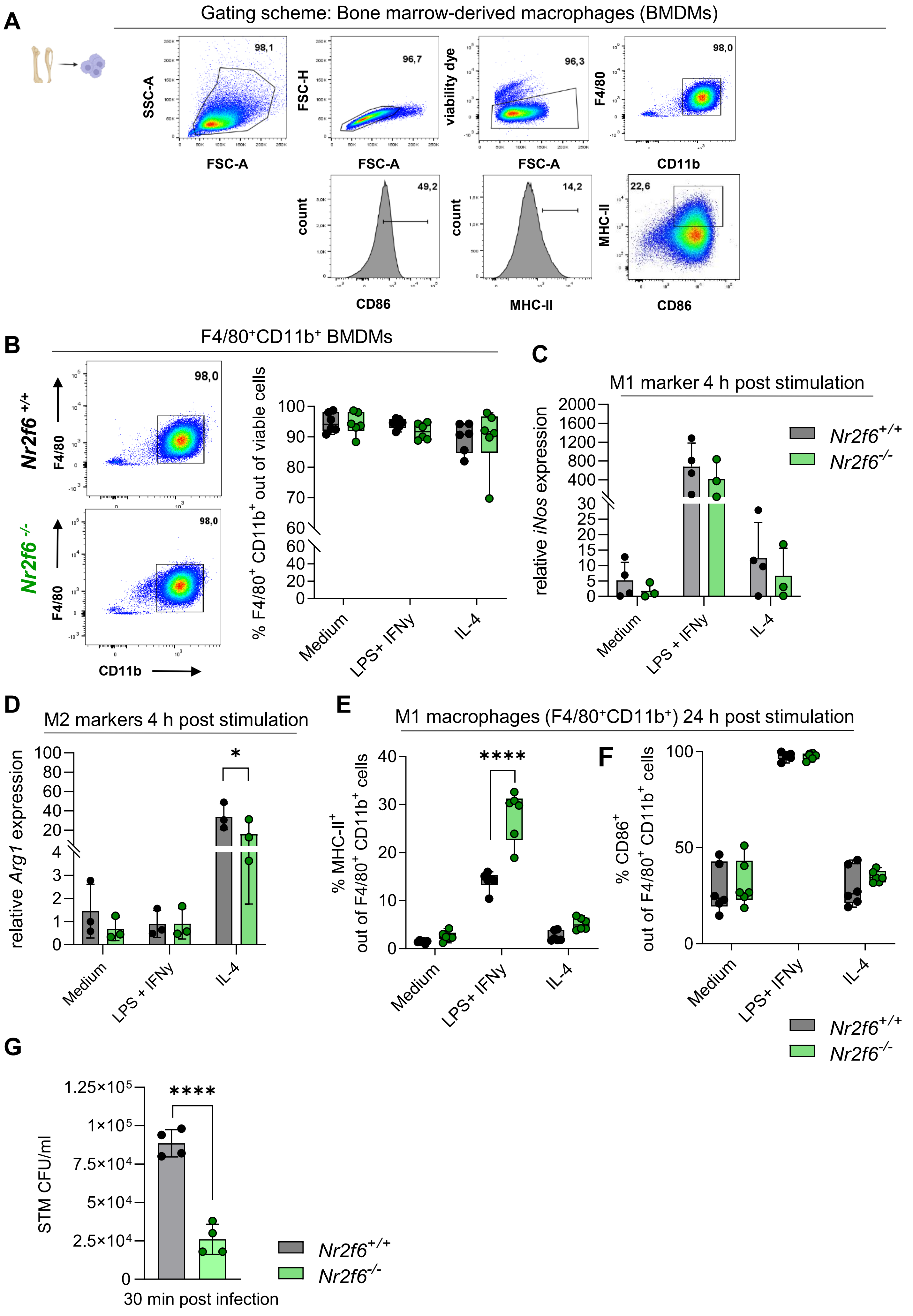
A
Gating scheme: Bone marrow-derived macrophages (BMDMs)
98,1
96,7
96,3
98,0
SSC-A
FSC-A
FSC-H
FSC-A
viability dye
FSC-A
F4/80
CD11b
49,2
14,2
22,6
count
CD86
count
MHC-II
MHC-II
CD86
B
F4/80⁺CD11b⁺ BMDMs
Nr2f6 +/+
Nr2f6 -/-
98,0
98,0
F4/80
F4/80
CD11b
% F4/80⁺ CD11b⁺ out of viable cells
Medium
LPS+ IFNy
IL-4
C
M1 marker 4 h post stimulation
relative iNos expression
Nr2f6+/+
Nr2f6-/-
Medium
LPS + IFNy
IL-4
D
M2 markers 4 h post stimulation
relative Arg1 expression
*
Medium
LPS + IFNy
IL-4
E
M1 macrophages (F4/80⁺CD11b⁺) 24 h post stimulation
% MHC-II⁺ out of F4/80⁺ CD11b⁺ cells
****
Medium
LPS+ IFNy
IL-4
F
% CD86⁺ out of F4/80⁺ CD11b⁺ cells
Medium
LPS+ IFNy
IL-4
Nr2f6+/+
Nr2f6-/-
G
STM CFU/ml
1.25×10⁵
1×10⁵
7.5×10⁴
5×10⁴
2.5×10⁴
0
****
Nr2f6+/+
Nr2f6-/-
30 min post infection

Supplemental Figure 3: Intrinsic loss of NR2F6 in BMDMs alters M1 and M2 differentiation and *Salmonella* Typhimurium phagocytosis *in vitro*.

(A) Dot plots and histograms showing the gating strategy to identify BMDMs ($F4/80^{+}CD11b^{+}$). Activated BMDMs were defined as $CD86^{+}$ and MHC-II^{+}. M1 BMDMs were defined as MHC-II^{+} $CD86^{+}$. **(B)** Representative dot-plots and quantification of BMDMs ($F4/80^{+}CD11b^{+}$) under resting, activated M1 (LPS+IFNγ) or M2 (IL-4) conditions of wild-type (*Nr2f6*$^{+/+}$) or *Nr2f6*-deficient BMDMs. **(C)** Quantification of mRNA expression of M1 (*iNos*) and **(D)** M2 (*Arg1*) markers of resting, M1 activated (LPS+IFNγ) or M2 (IL-4) activated conditions of wild-type (*Nr2f6*$^{+/+}$) or *Nr2f6*-deficient BMDMs. **(E)** Quantification of frequencies of MHC-II and **(F)** CD86 positive cells within BMDMs under resting, M1 or M2 wild-type (*Nr2f6*$^{+/+}$) or *Nr2f6*-deficient (*Nr2f6*$^{-/-}$) BMDMs 24 h post differentiation. **(G)** Quantification of colony-forming units (CFUs) from wild-type (*Nr2f6*$^{+/+}$) or *Nr2f6*-deficient (*Nr2f6*$^{-/-}$) *Salmonella* Typhimurium infected BMDMs 30 min post infection. Representative data shown are from at least two independent experiments with n = 2-3 per group and experiment. Only M2 stimulation was only performed one with n=3. Each dot represents the data of an individual mouse-derived BMDM culture. Results are shown as median ± IQR with whiskers from min. to max. (B+E) or mean ± SEM (C,D,G), or. The Shapiro-Wilk test evaluated the normality of data. Asterisks indicate statistically significant differences between genotypes calculated using the Student's *t*-test or Mann-Whitney *U* test for non-parametric data. A p-value < 0.05 was considered statistically significant. *$p < 0.05$; ****$p < 0.0001$.

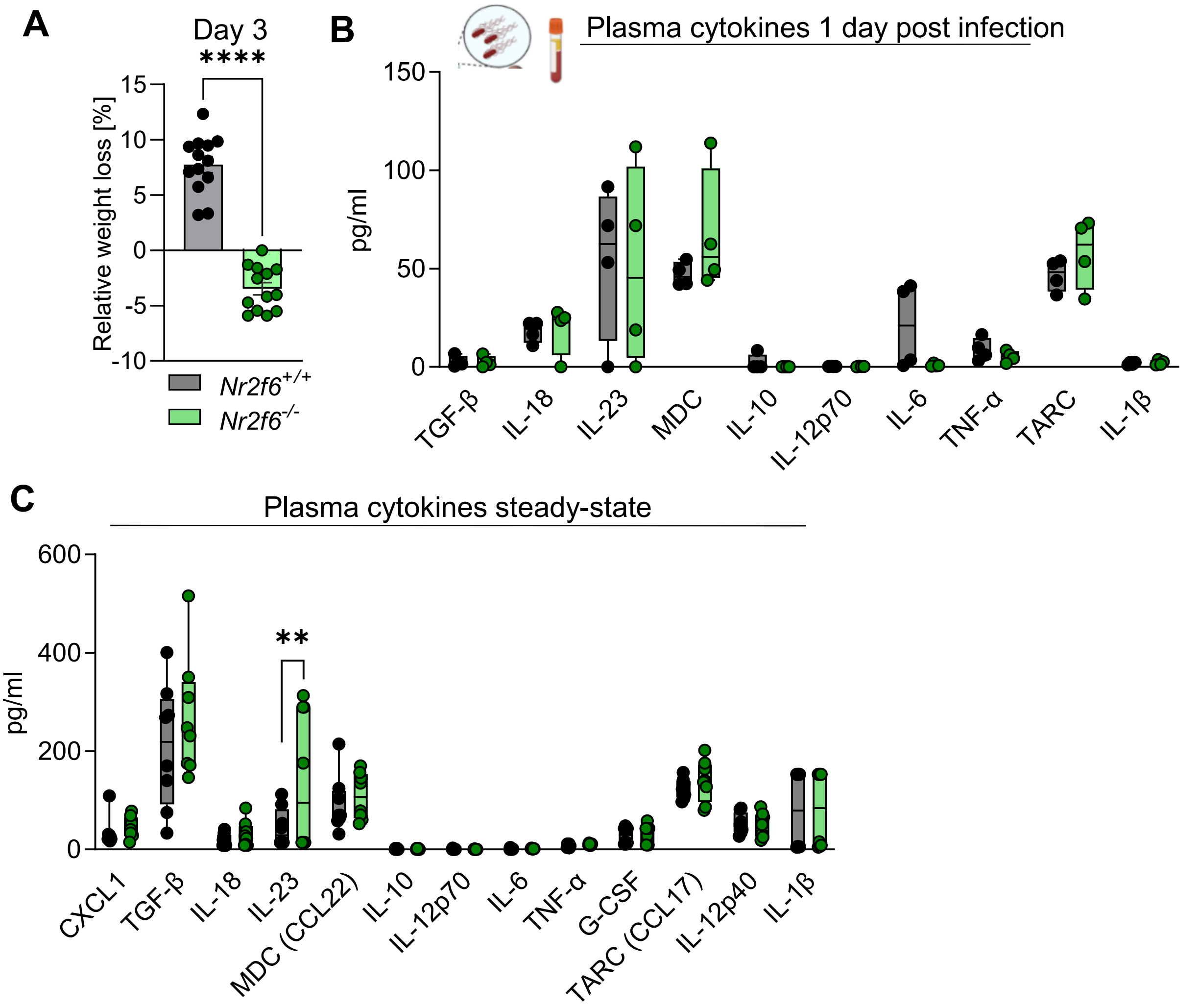

Supplemental Figure 4: Loss of NR2F6 protects mice from *Salmonella* Typhimurium infection.

(A) Relative weight loss of wild-type (*Nr2f6*$^{+/+}$) or *Nr2f6*-deficient (*Nr2f6*$^{-/-}$) mice 3 days post *Salmonella* Typhimurium infection. **(B)** Plasma cytokine and chemokine levels of wild-type (*Nr2f6*$^{+/+}$) or *Nr2f6*-deficient (*Nr2f6*$^{-/-}$) mice 1 day post *Salmonella* Typhimurium infection. **(C)** Plasma cytokine and chemokine levels of healthy wild-type (*Nr2f6*$^{+/+}$) or *Nr2f6*-deficient (*Nr2f6*$^{-/-}$) mice.

Representative data shown are from at least two independent experiments with $n = 2$ per group and experiment. Each dot represents the data of an individual mouse. Results are shown as mean ± SEM (A), or median ± IQR with whiskers from min. to max. The Shapiro-Wilk test evaluated the normality of data. Asterisks indicate statistically significant differences between genotypes calculated using the Student's *t*-test or Mann-Whitney *U* test for non-parametric data. A *p*-value < 0.05 was considered statistically significant. **$p < 0.01$, ****$p < 0.001$.

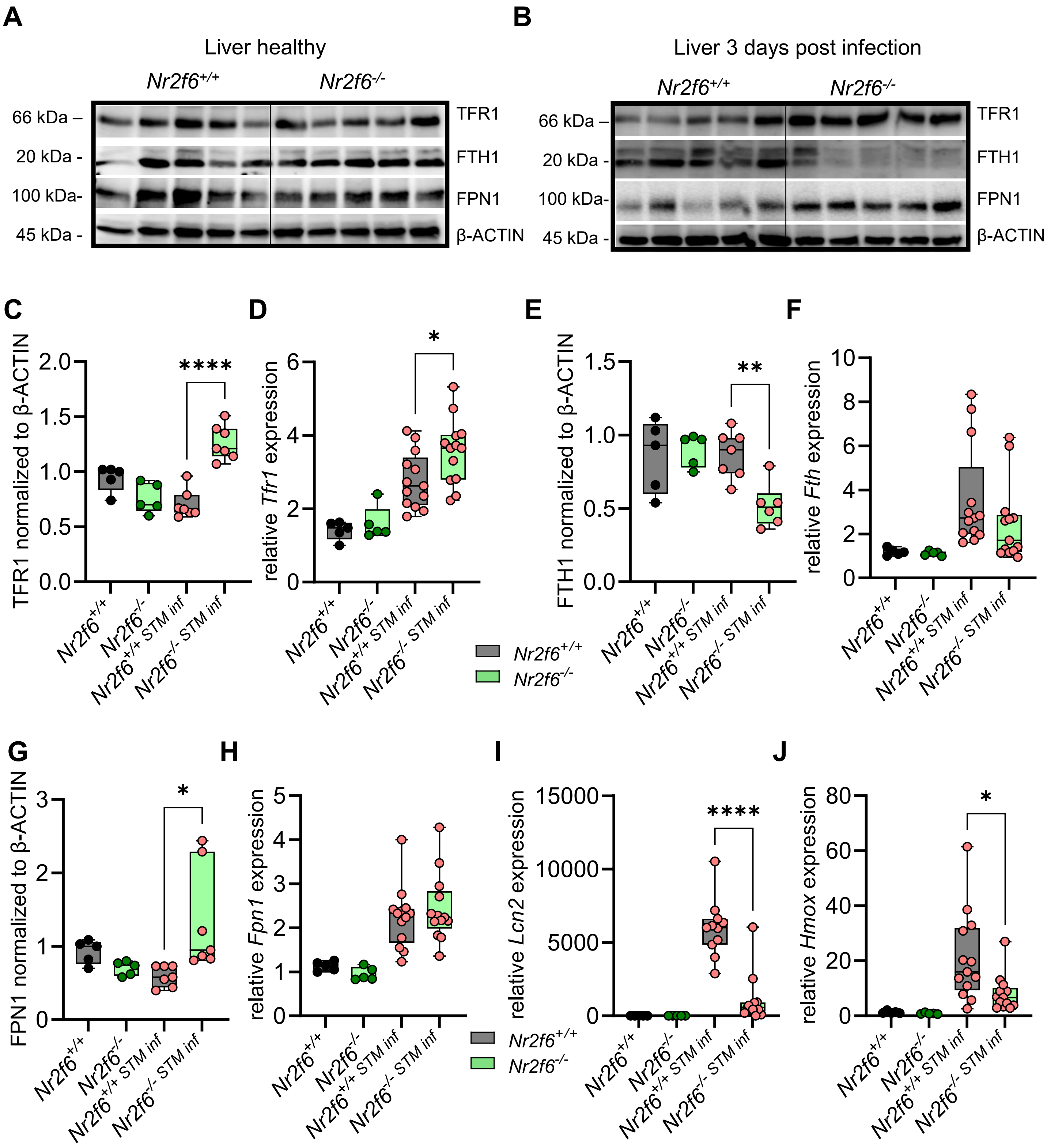
A
Liver healthy
Nr2f6+/+
Nr2f6-/-
66 kDa –
20 kDa -
100 kDa-
45 kDa -
TFR1
FTH1
FPN1
β-ACTIN
B
Liver 3 days post infection
C
TFR1 normalized to β-ACTIN
****
D
relative Tfr1 expression
*
E
FTH1 normalized to β-ACTIN
**
F
relative Fth expression
G
FPN1 normalized to β-ACTIN
*
H
relative Fpn1 expression
I
relative Lcn2 expression
****
J
relative Hmox expression
*
Nr2f6+/+ STM inf
Nr2f6-/- STM inf

Supplemental Figure 5: Liver iron transport proteins in healthy and *Salmonella* infected *Nr2f6*-deficient mice.

(A) Representative protein abundance of the transferrin receptor1 (TFR1), ferritin (FTH1), ferroportin1 (FPN1), and β-ACTIN in the liver of healthy, or **(B)** 3 days post *Salmonella* Typhimurium infection of wildtype (*Nr2f6*$^{+/+}$) or *Nr2f6*-deficient (*Nr2f6*$^{-/-}$) mice. **(C)** Representative protein abundance of TFR1 normalized to β-ACTIN. **(D)** Quantification of fold change *Tfr1* mRNA expression relative to *Hprt* in the liver of healthy or 3 days post *Salmonella* Typhimurium infection of wildtype (*Nr2f6*$^{+/+}$) or *Nr2f6*-deficient (*Nr2f6*$^{-/-}$) mice. **(E)** Representative protein abundance of FTH1 normalized to β-ACTIN , **(F)** quantification of fold change *Fth1* mRNA expression relative to *Hprt* in the liver of healthy or 3 days post *Salmonella* Typhimurium infection of wildtype (*Nr2f6*$^{+/+}$) or *Nr2f6*-deficient (*Nr2f6*$^{-/-}$) mice. **(G)** Representative protein abundance of FPN1 normalized to β-ACTIN. **(H)** Quantification of fold change *Fpn1* mRNA expression relative to *Hprt* in the liver of healthy or 3 days post *Salmonella* Typhimurium infection of wildtype (*Nr2f6*$^{+/+}$) or *Nr2f6*-deficient (*Nr2f6*$^{-/-}$) mice. **(I)** Quantification of fold change *Lcn2* and **(J)** *Hmox* mRNA expression relative to *Hprt* in the liver of healthy or 72 h post *Salmonella* Typhimurium infection of wildtype (*Nr2f6*$^{+/+}$) or *Nr2f6*-deficient (*Nr2f6*$^{-/-}$) mice. Representative data shown are from at least two independent experiments with *n* = 2-3 per group and experiment, total *n* = 13/9, and *n* = 5/5 (for steady state) (*Nr2f6*$^{+/+}$)/(*Nr2f6*$^{- /-}$). Each dot represents the data from one individual mouse. Quantification of mRNA fold change was calculated relative to one uninfected wild-type (*Nr2f6*$^{+/+}$) set as 1. Results are shown as median ± IQR with whiskers from min. to max. The Shapiro-Wilk test evaluated the normality of data. Asterisks indicate statistically significant differences between genotypes calculated using the one-way Anova. A *p*-value < 0.05 was considered statistically significant, **0.01, ***0.001, ****.

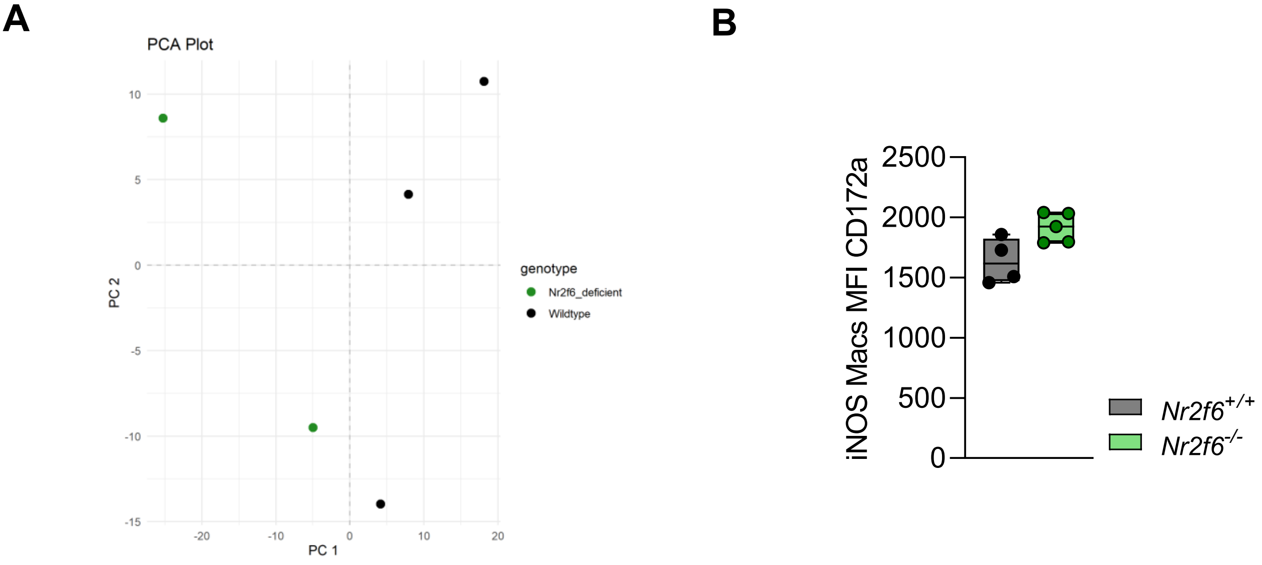

Supplemental Figure 6: RNA-Seq of *Nr2f6*-deficient red pulp macrophages and verification of target genes via flow cytometry.

Principal component analysis (PCA) of sorted splenic red pulp macrophages from three healthy wild-type (*Nr2f6*$^{+/+}$) and two *Nr2f6*-deficient (*Nr2f6*$^{-/-}$) mice.

(B) Quantification of the Sirpα (CD172a) MFI of wild-type (*Nr2f6*$^{+/+}$) or *Nr2f6*-deficient iNOS^{+} splenic macrophages of wild-type (*Nr2f6*$^{+/+}$) or *Nr2f6*-deficient *Salmonella* Typhimurium infected mice.

Representative data shown (A) are from one experiments with n = 3/2 per group and experiment. Representative data shown (B) are from one of two independent experiments with n = 2-3 per group and experiment, total n = 4/5, (*Nr2f6*$^{+/+}$)/(*Nr2f6*$^{-/-}$). Each dot represents the data from one individual mouse. Results are shown as median ± IQR with whiskers from min. to max. The Shapiro-Wilk test evaluated the normality of data. Asterisks indicate statistically significant differences between genotypes calculated using the Mann-Whitney U test for non-parametric data. A p-value < 0.05 was considered statistically significant. *p < 0.05,**0.01, ***0.001.

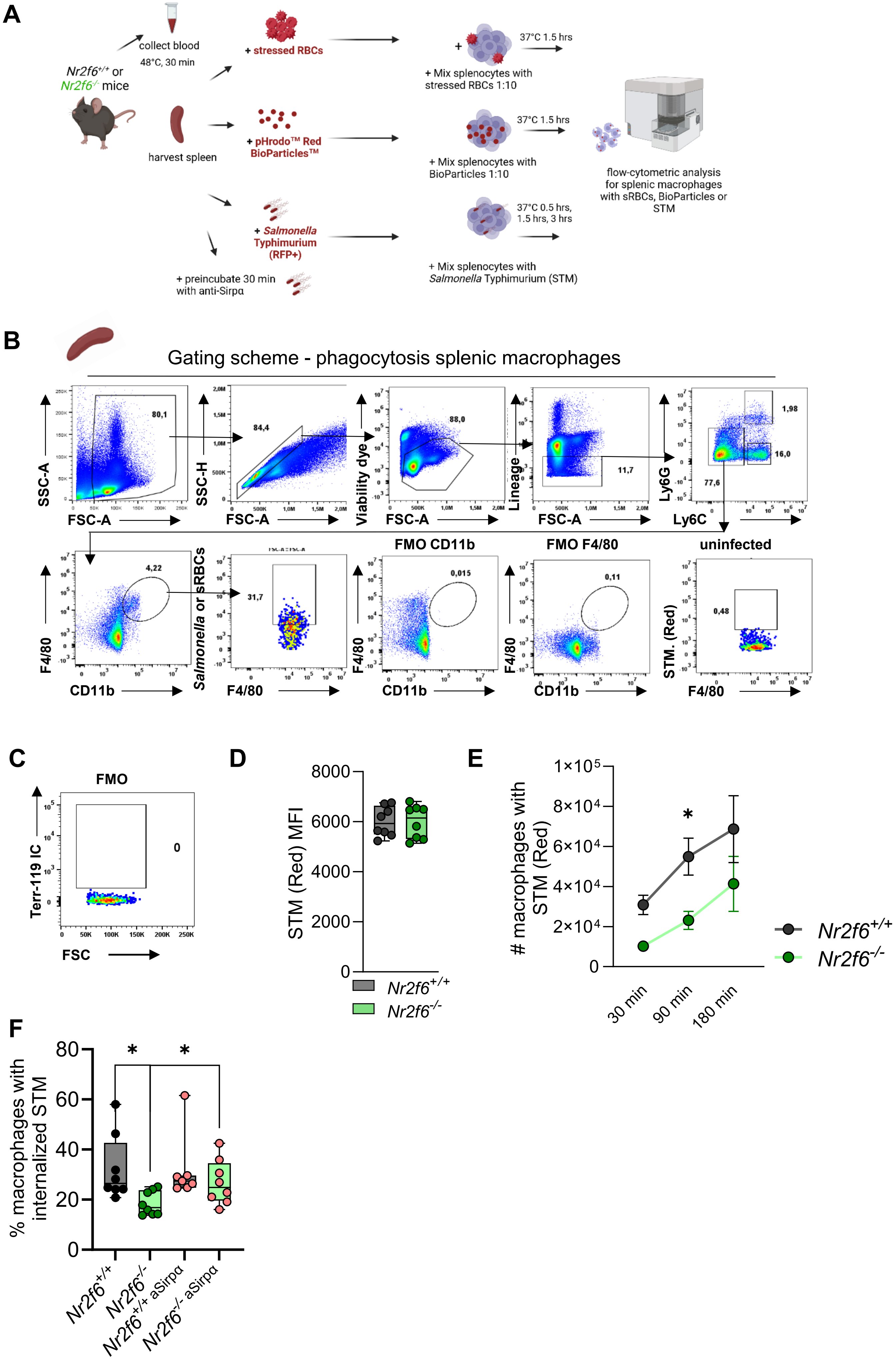
A
collect blood
48°C, 30 min
Nr2f6+/+ or
Nr2f6-/- mice
harvest spleen
+ stressed RBCs
+ pHrodo™ Red BioParticles™
+ Salmonella Typhimurium (RFP+)
+ preincubate 30 min with anti-Sirpα
37°C 1.5 hrs
+ Mix splenocytes with stressed RBCs 1:10
37°C 1.5 hrs
+ Mix splenocytes with BioParticles 1:10
37°C 0.5 hrs, 1.5 hrs, 3 hrs
+ Mix splenocytes with Salmonella Typhimurium (STM)
flow-cytometric analysis for splenic macrophages with sRBCs, BioParticles or STM
B
Gating scheme - phagocytosis splenic macrophages
SSC-A
FSC-A
80,1
SSC-H
84,4
Viability dye
88,0
Lineage
11,7
Ly6G
Ly6C
1,98
16,0
77,6
F4/80
CD11b
4,22
Salmonella or sRBCs
31,7
FMO CD11b
0,015
FMO F4/80
0,11
uninfected
STM. (Red)
0,48
C
FMO
Terr-119 IC
FSC
0
D
STM (Red) MFI
8000
6000
4000
2000
0
Nr2f6+/+
Nr2f6-/-
E
macrophages with STM (Red)
1×10^5
8×10^4
6×10^4
4×10^4
2×10^4
0
*
30 min
90 min
180 min
Nr2f6+/+
Nr2f6-/-
F
% macrophages with internalized STM
80
60
40
20
0
*
*
Nr2f6+/+
Nr2f6-/-
Nr2f6+/+ aSirpα
Nr2f6-/- aSirpα

Supplemental Figure 7: Phagocytic responses in *Nr2f6*-deficient mice *in vitro*.

(A) Scheme depicting the experimental procedure of *Nr2f6*$^{+/+}$ or *Nr2f6*$^{-/-}$ splenic harvest following incubation of splenocytes with either stressed red blood cells (sRBCs), pHrodo Zymosan BioParticles or *Salmonella* Typhimurium (STM), with or without 30 minutes of anti-Sirpα (aSirpα) treatment, for the indicated time points and subsequent flow cytometric analysis (Created with BioRender.com). **(B)** Gating strategy to identify sRBC/BioParticles or *Salmonella* Typhimurium phagocytosis of splenic macrophages defined as live single-cells, Lin^{-}Ly6G^{-},Ly6C^{-}CD11b^{int}F4/80^{+} and further examined on sRBCc (Ter119 intracellular), phagolysosomal specific BioParticle, or *Salmonella* Typhimurium uptake of healthy wild-type (*Nr2f6*$^{+/+}$) or *Nr2f6*-deficient (*Nr2f6*$^{-/-}$) mice, FMO controls for CD11b, F4/80 and one uninfected sample are shown. **(C)** FMO controls for Terr119 is shown. **(D)** MFI of *Salmonella* Typhimurium uptake into splenic macrophages of wild-type (*Nr2f6*$^{+/+}$) or *Nr2f6*-deficient spleens. **(E)** Quantification of total splenic macrophages cell numbers containing phagocytosed *Salmonella* Typhimurium 30, 90, and 180 minutes after infection of wild-type (*Nr2f6*$^{+/+}$) or *Nr2f6*-deficient spleens. **(F)** Quantification of *Salmonella* Typhimurium cell uptake after 30 minutes of anti-Sirpα pre-incubation and 90 minutes after infection into splenic macrophages of wild-type (*Nr2f6*$^{+/+}$) or *Nr2f6*-deficient spleens. Representative data shown are from n = 8 per group. Each dot represents the data of an individual mouse-derived spleen culture. Results are shown as median ± IQR with whiskers from min. to max (**D+F**) or mean ± SEM (**E**). The Shapiro-Wilk test evaluated the normality of data. Asterisks indicate statistically significant differences between genotypes calculated using the Student's *t*-test or Mann-Whitney *U* test for non-parametric data.
